# Both Las17-binding sites on Arp2/3 complex are important for branching nucleation and assembly of functional endocytic actin networks in *S. cerevisiae*

**DOI:** 10.1016/j.jbc.2024.105766

**Published:** 2024-02-16

**Authors:** Heidy Y. Narvaez-Ortiz, Michael J. Lynch, Su-Ling Liu, Adam Fries, Brad J. Nolen

**Affiliations:** Department of Chemistry and Biochemistry, Institute of Molecular Biology, University of Oregon, Eugene, Oregon, USA

**Keywords:** actin, Arp2/3 complex, nucleation, WASP, Las17, endocytosis

## Abstract

Arp2/3 complex nucleates branched actin filaments that drive membrane invagination during endocytosis and leading-edge protrusion in lamellipodia. Arp2/3 complex is maximally activated *in vitro* by binding of a WASP family protein to two sites—one on the Arp3 subunit and one spanning Arp2 and ARPC1—but the importance of each site in the regulation of force-producing actin networks is unclear. Here, we identify mutations in budding yeast Arp2/3 complex that decrease or block engagement of Las17, the budding yeast WASP, at each site. As in the mammalian system, both sites are required for maximal activation *in vitro*. Dimerization of Las17 partially restores activity of mutations at both CA-binding sites. Arp2/3 complexes defective at either site assemble force-producing actin networks in a bead motility assay, but their reduced activity hinders motility by decreasing actin assembly near the bead surface and by failing to suppress actin filament bundling within the networks. While even the most defective Las17-binding site mutants assembled actin filaments at endocytic sites, they showed significant internalization defects, potentially because they lack the proper architecture to drive plasma membrane remodeling. Together, our data indicate that both Las17-binding sites are important to assemble functional endocytic actin networks in budding yeast, but Arp2/3 complex retains some activity *in vitro* and *in vivo* even with a severe defect at either Las17-binding site.

Arp2/3 complex is a seven-subunit oligomeric protein that regulates the assembly of the actin cytoskeleton by nucleating branched actin filaments (1, 2). The complex is conserved from yeast to metazoans (1), and the branched actin networks it assembles play critical roles in processes ranging from endocytosis and cellular motility to meiosis and DNA damage repair (3, 4). By tightly regulating the activity of Arp2/3 complex, cells assemble branched actin networks at the right time and place in the cell to drive these diverse cellular processes.

Because it has little or no intrinsic activity, Arp2/3 complex depends on nucleation promoting factors (NPFs) to trigger nucleation (1). WASP proteins comprise the largest class of NPFs, and while diverse in their N-terminal domains, all WASP family NPFs harbor conserved C-terminal VCA (Verprolin homology, Central, Acidic) segments, the minimal region of WASP sufficient for activation of the complex (5, 6, 7, 8). Biochemical and structural data show that the WASP CA binds directly to Arp2/3 complex, and the V segment (also called WH2, WASP homology 2) recruits actin monomers to the complex (7, 9, 10, 11). The mechanism of WASP-mediated activation of Arp2/3 complex involves multiple sets of interactions; not only must WASP bind the complex and recruit actin monomers but also the complex must bind ATP and the side of pre-existing actin filament (12, 13, 14). How Arp2/3 complex integrates signals from all these activating factors is still an important open question.

WASP–CA binds to two sites on Arp2/3 complex (9, 10, 15, 16, 17), and only recently has structural information about how WASP engages the two sites become available (9, 10, 18). One CA binds Arp2 and ARPC1, inserting its C region helix into the Arp2 barbed end groove and a conserved tryptophan from its A segment into a shallow pocket on the ARPC1 surface (Fig. 1*A*). A second CA binds Arp3, with its C segment forming a helix that engages the Arp3 barbed end groove and its A segment inserting a conserved tryptophan into a pocket at the interface of subdomains 3 and 4 (Fig. 1*A*). Biochemical experiments showed that N-WASP and WAVE, two human WASP family proteins, bind to the Arp2–ARPC1 site ∼5–60-fold tighter than the Arp3 site (16, 17). By engaging the two sites, WASP stimulates a large structural rearrangement—the short pitch conformational change—that brings the Arp2 and Arp3 subunits into a filament-like arrangement. Bound WASP also recruits the first actin monomers to the barbed ends of Arp2 and Arp3 for incorporation into the new (daughter) filament (9, 15, 19, 20, 21). The precise contribution of CA engagement at each site to these activating steps is unclear. For instance, some experiments suggest that only the Arp2–ARPC1 site is involved in triggering the short pitch conformational change (18), whereas others suggest that both sites contribute (20).

**Figure 1:**
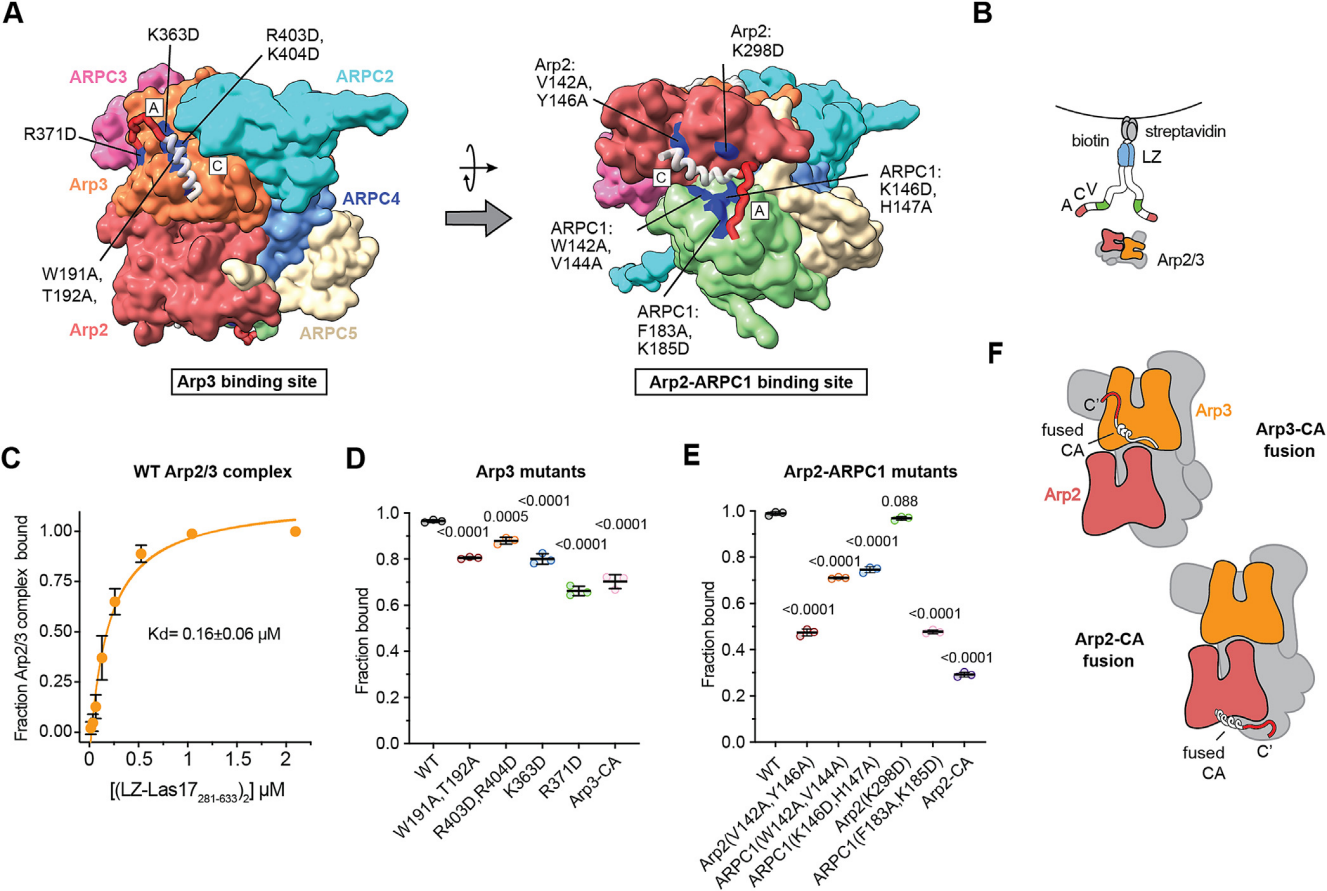
**Mutations to the Arp3 or Arp2–ARPC1 CA-binding sites cause defects in Las17 binding.***A*, surface representation of a homology model of ScArp2/3 complex with bound Las17–CA showing location of mutations at the Arp3 and Arp2–ARPC1 sites. Binding of Las17–CA was modeled based on both crosslinking/mass spectrometry and cryo-EM data (9, 10). The C and A segments of Las17 are labeled and colored *gray* and *red*, respectively. *B*, schematic of the supernatant depletion assays showing the construct of Las17 used to pulldown the WT or CA-binding site mutant complexes. *C*, binding isotherm for LZ-Las17_281–633_ generated using the supernatant depletion assay in PC buffer. Reaction was run in triplicate with the same protein stocks. Concentration on *x*-axis is the concentration of the LZ-Las17_281–633_ dimer. Error bars represent standard deviation. Error bars for last two data points were approximately the same size as the data points, so were omitted. *D* and *E*, plot of the fraction of Arp2/3 complex bound in supernatant depletion reactions containing 0.6 μM (LZ-Las17_281–633_)__2__. Statistical significance was measured with an ordinary one-way ANOVA with *p* values for comparisons to WT indicated. Data points represent technical replicates. Error bars represent standard deviation. *F*, diagram of the Arp2 and Arp3 CA fusion complexes, in which the CA of N-WASP is fused to the N terminus of the Arp2 or Arp3 subunit.

Measurements of Arp2/3 complex activity in bulk fluorescence–based actin polymerization assays showed that both CA-binding sites are required for maximal WASP-mediated activation in human Arp2/3 complex (9). However, for several reasons, bulk assays may not fully reflect the importance of the interactions between WASP and Arp2/3 complex in regulating actin networks in cells. First, the WASP–Arp2/3 complex interaction influences the actin network beyond Arp2/3 complex activation. For instance, the prenucleation interaction of WASP and filament-bound Arp2/3 complex connects membranes to polymerizing actin networks (22, 23). This connection influences actin filament network architectures by counteracting the polymerization force and compressing the network (22). It may also be involved in a negative feedback mechanism in which NPFs are removed from the membrane *via* the connection of WASP with an inward-treadmilling actin network (24). These secondary roles of the WASP–Arp2/3 interaction are absent in bulk fluorescence–based actin polymerization assays. In addition, such assays may not accurately report on the level of Arp2/3 complex activity in a cell, either because of limitations in replicating *in vivo* conditions (*e.g.*, protein concentrations, buffer conditions) or because of the influence of other cellular proteins on activity. For these reasons, probing the importance of each site in reconstituted motility systems and *in vivo* is critical to understand the molecular basis for WASP-mediated regulation of branched actin networks.

Indeed, biochemical data already indicate that at least a subset of CA-binding site mutations that cause significant defects *in vitro* might be rescued in cells. Specifically, Zimmet *et al.* (9) found that *in vitro*, the decreased activity of an Arp3 CA-binding site mutation could be partially restored by dimerizing WASP. They hypothesized that dimerization allows the NPF to recruit an actin monomer to the Arp3 site even if the CA cannot engage it. In cells, WASP proteins are thought to cluster on the membrane at high densities, making it likely that they activate Arp2/3 complex as oligomers (25, 26, 27). This raises the possibility that cellular WASP dimers could rescue defects at the Arp3 site, making it possible for the complex to function with only the Arp2–ARPC1 binding site.

In budding yeast, Arp2/3 complex assembles branched actin networks that drive internalization of endocytic cargoes during clathrin-mediated endocytosis (28, 29, 30). Actin filaments are nucleated at the membrane to provide pushing forces that create a ∼0.1 to 0.2 μm tubule (31, 32, 33). The center of mass (centroid) of filaments and associated proteins slowly moves inward by ∼0.1 micron as the membrane tubule elongates (32). Scission then occurs, and the actin-coated vesicle moves rapidly away from the cell edge (33). Several NPFs are present at endocytic sites in budding yeast, including Las17 (the budding yeast WASP), type 1 myosins (myo3 and myo5), Abp1, and Pan1 (34). Each of these NPFs harbor CA or A sequences that can bind to Arp2/3 complex to trigger branching nucleation, though Las17 and type 1 myosins have the strongest NPF activity (34, 35). Mutations of the Arp2/3-binding regions of these NPFs, alone or in combination, showed they have distinct but overlapping roles in regulating endocytic actin assembly (36, 37). Quantitative analysis of the phenotypes of the NPF mutations led to a negative feedback model, in which assembly of a functional endocytic network by NPF-activated Arp2/3 complex leads to the removal of NPFs from the membrane by the actin network (24). While this model helps explain how the bursts of actin polymerization can be generated during endocytosis, it has not been extensively challenged.

Here, we identify point mutations in budding yeast Arp2/3 complex that completely or nearly completely block engagement of Las17 at each CA-binding site. These mutants allow us to probe the role of WASP binding at each site in bulk actin assembly assays, reconstituted bead motility assays, and at sites of endocytosis in budding yeast. They also allow us to test the negative feedback model for NPF removal from endocytic sites.

## Results

### Generation of mutant Arp2/3 complexes and Las17 for biochemical assays

We designed a total of four mutations in the Arp3 site and five mutations in the Arp2–ARPC1 site based on a crosslinking/mass spectrometry model of Las17 CA bound to Arp2/3 complex (10). This model shows Las17 bound in locations nearly equivalent to those observed in the cryo-EM structure of N-WASP CA bound to human Arp2/3 complex (9), with the exception of a shift in the position of the C-helix at Arp2–ARPC1 (Fig. 1*A*). The mutations targeted both the C and A binding regions within each of the two sites.

To generate mutant complexes, we expressed mutated Arp3, Arp2, or ARPC1 under its native promoter in an exogenous locus and knocked out the corresponding WT gene. To facilitate purification, the mutations were generated in budding yeast strains expressing the ARPC2 subunit with C-terminal tandem 12xHis and TwinStrep tags (38). We purified the complex using both Strep-Tactin and nickel–nitrilotriacetic acid (Ni–NTA) affinity columns (Fig. S1*A*), and found that in pyrene actin polymerization assays, it had slightly higher activity than untagged Arp2/3 complex purified with a glutathione-*S*-transferase (GST)-WASP-VCA affinity column (Fig. S1*B*). The activity of the tandem-tagged Arp2/3 complex was consistent across multiple preparations (Fig. S1*C*). To ensure that the influence of the mutations in biochemical assays accurately reflected their influence on Arp2/3 complex activity *in vivo*, we carried out all *in vitro* assays in this study in a buffer designed to mimic the composition of the yeast cytoplasm (physiological condition [PC] buffer), unless otherwise noted (39, 40). Notably, this buffer has a higher ionic strength than the buffer typically used for measuring actin polymerization (Fig. S1*D*).

While most biochemical experiments with Las17 have used the C-terminal VCA segment, the minimal region of Las17 sufficient to activate Arp2/3 complex, regions outside the VCA segment can influence the ability of Las17 to activate Arp2/3 complex (41). Therefore, for the biochemical experiments we report here, we used a construct including residues 281 to 633 of Las17, which contains most of central polyproline segment of Las17 and the entire C-terminal VCA segment (Fig. S1*E*). Large quantities of this construct (Las17_281–633_) could be expressed and purified from *Escherichia coli* (Fig. S1, *E* and *F*). Las17_281–633_ activates Arp2/3 complex significantly more potently than the VCA segment alone in the low salt buffer, as previously reported for full-length Las17 purified from yeast (Fig. S1*G*) (41). However, Las17_281–633_ did not activate Arp2/3 complex more potently than Las17-VCA in PC buffer (Fig. S1*H*). Las17_281–633_ had no Arp2/3-independent actin filament nucleation activity in PC buffer (Fig. S1, *I* and *J*), despite reports that similar Las17 constructs nucleate filaments in a standard low salt actin polymerization buffer (42, 43).

### Mutations to the Arp3 or Arp2–ARPC1 CA-binding sites cause defects in Las17 binding

To measure their binding affinity for Las17, we depleted Arp2/3 complexes from a solution using streptavidin-coated beads bound to biotinylated Las17_281–633_ and measured the Arp2/3 complex remaining in solution (Figs. 1*B* and S2). Previous biochemical data show that dimerization increases the potency of WASP proteins (44), and Las17 and other WASP family proteins are thought to act as membrane-bound oligomers in cells (25, 26, 27). Therefore, we tagged the Las17_281–633_ construct at its N terminus with a leucine-zipper domain to ensure that it engaged Arp2/3 complex as a dimer in binding assays (Fig. 1*B*). Based on this assay, the affinity of the LZ-Las17_281–633_ construct for WT Arp2/3 complex is 0.16 μM in PC buffer (Figs. 1C and S2). We next tested each of the Arp3 site mutants at a single concentration (0.6 μM) of (LZ-Las17_281–633_)_2_. Overall, none of the Arp3 site mutations had a dramatic influence on binding (Fig. 1*D*). The most defective mutant, Arp3(R371D), was in the A-site and showed a 30% reduction in binding compared with WT Arp2/3 complex. The other mutants in the C site or A site on Arp3 showed a binding reduction of 10 to 20%.

Mutations at the Arp2–ARPC1 site also impaired Las17 binding, sometimes more severely than the Arp3 mutants. For instance, Arp2(V142A, Y146A) and ARPC1(F183A, K185D) showed a 50% reduction in binding at 0.6 μM (LZ-Las17_281–633_)_2_ compared with the WT complex (Fig. 1*E*). The relative decreases in affinity for the mutants are less than predicted based on the affinities previously measured for N-WASP-VCA from *Saccharomyces cerevisiae* lysate (10). The lower sensitivity of binding affinity to the mutations we report here may be due to the different WASP family protein used for the assay, the use of purified proteins *versus* lysate, or different buffer conditions.

### Comparison of point mutants to Arp2–CA or Arp3–CA fusion complexes

The supernatant depletion–binding assay measures the affinity of dimeric Las17 for the complex, so even if one site is completely ablated by mutation, the other site can engage WASP and pull down the complex. Therefore, to provide more information about the relative effect of the point mutations at the individual sites, we compared the affinity of LZ-Las17_281–633_ to previously designed chimeras in which the CA segment of N-WASP is fused to the C terminus of either Arp3 or Arp2 (Fig. 1*F*) (19, 20). These chimeras block crosslinking of free monomeric WASP at the site engaged by the fused CA segment (20). Therefore, the CA fusions show the fraction of the complex bound to LZ-Las17_281–633_ when one site is completely or strongly blocked.

As anticipated, the CA fusions decreased binding, with Arp3–CA and Arp2–CA pulling down 73% and 30% of the complex at 0.6 μM (LZ-Las17_281–633_)_2_, respectively (Fig. 1, *D* and *E*). Importantly, the Arp3–CA fusion showed a comparable decrease in Las17 binding as the weakest binding point mutant at that site, Arp3(R371D). These data suggest that the Arp3(R371D) point mutation strongly or completely blocks Las17 binding at that site. At the Arp2–ARPC1 site, the Arp2(V142A, Y146A) and ARPC1(F183A, K185D) mutants were less impaired than the Arp2–CA fusion, each showing ∼50% reduction in binding. Therefore, while these mutations are relatively potent at disrupting binding of the Las17 dimer, they appear to do so by moderately (but not completely) blocking Las17 binding to the Arp2–ARPC1 site.

### Mutations to the Arp3 or Arp2–ARPC1 CA-binding sites cause defects in Myo5 binding

Each of the NPFs (Myo3, Myo5, Abp1, Pan1, and Crn1) in *S. cerevisiae* harbor CA or A sequences homologous to those in Las17 (35, 36, 45), suggesting they bind to the same sites as Las17. Biochemical and genetic data suggest that Las17 and Myo3/5 are the most potent NPFs (35). Therefore, we tested binding of the CA segment of one of two myosins, Myo5, to a subset of the CA-binding site mutants in Arp2/3 complex. We found that the dimerized Myo5 CA segment binds with similar affinity as Las17 CA (0.13 ± 0.04 μM) (Fig. S3). Arp2/3 complex mutations that caused defects in Las17 binding also caused defects in Myo5 CA binding, indicating the binding interactions are made with similar sets of residues (Figs. 1 and S3). However, the relative magnitudes of the binding defects for the two NPFs were not well correlated, suggesting that there are differences in the precise mode of binding (Fig. S3).

### Mutations in either CA-binding site cause defects but do not completely block Arp2/3 complex activation

**Figure 2:**
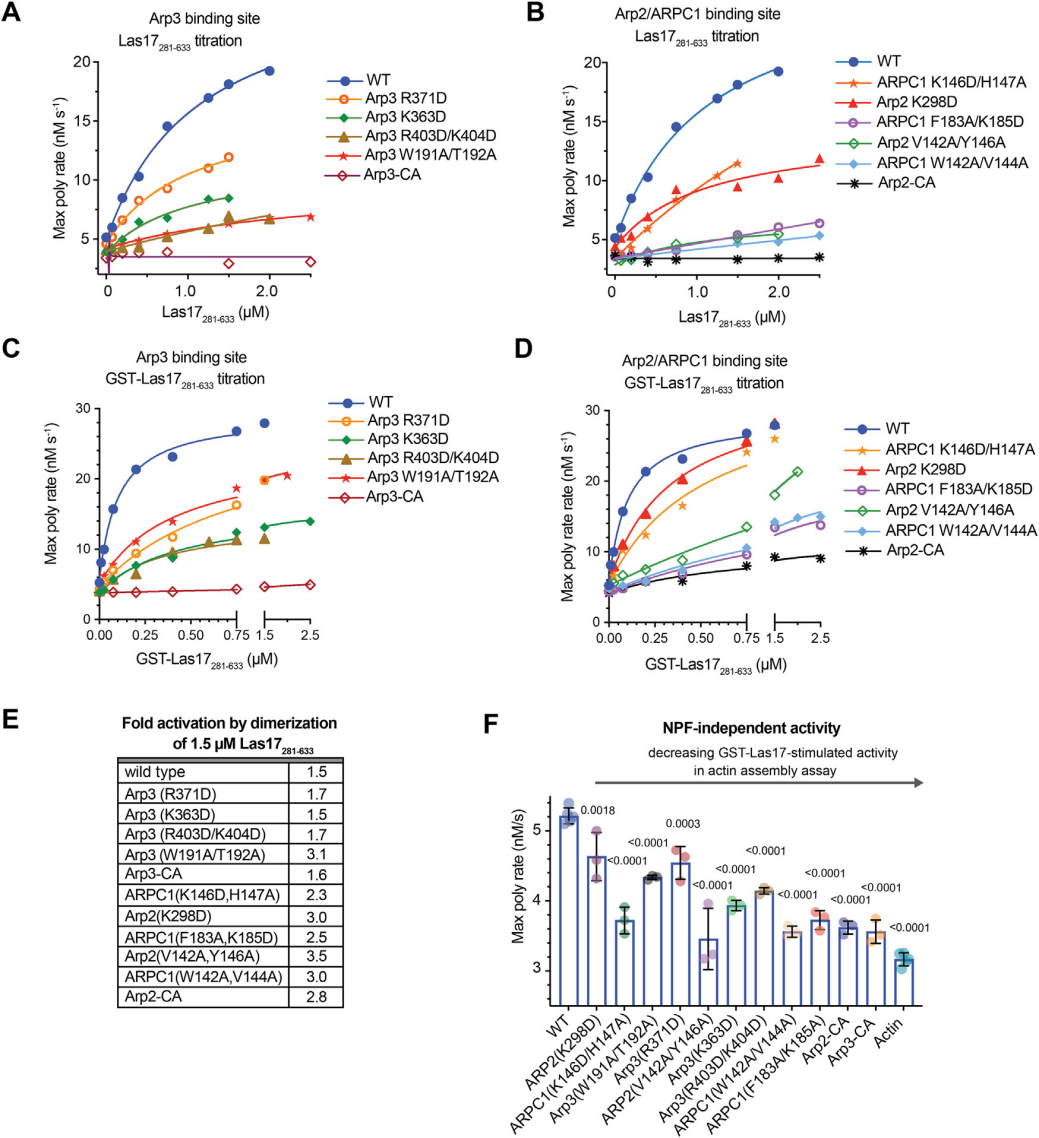
**Mutations in either CA-binding site cause defects in Arp2/3 complex activation.***A*, plot of the maximum polymerization rate *versus* Las17_281–633_ concentration for reactions containing 20 nM Arp2/3 complex (WT or Arp3 site mutant), 3 μM 15% pyrene actin, and the indicated concentrations of Las17_281–633_. *B*, identical to *A*, except the activity of the WT complex is compared with the Arp2–ARPC1 site mutants. *C*, identical to *A*, except WT and mutant complexes were titrated with GST-Las17_281–633_. The concentrations on the *x*-axis indicate the concentration of GST-Las17_281–633_ monomers. *D*, identical to *C*, except WT and mutant complexes are titrated with GST-Las17_281–633_. *E*, fold increased activation of Arp2/3 complexes upon dimerization of 1.5 μM Las17_281–633_. *F*, Maximum polymerization rate of 20 nM of WT and mutant Arp2/3 complexes determined from time courses of pyrene actin polymerization as in (*A*) but without NPF. Statistical significance was measured with an ordinary one-way ANOVA with indicated *p* values from comparison to WT calculated with a Dunnet’s test. Data points represent technical replicates of the assay. Error bars represent standard deviation. NPF, nucleation promoting factor.

To determine how the mutations influence the activity of the complexes, we tested their ability to nucleate filaments in pyrene actin polymerization assays. Despite their relatively mild binding defects, mutations in the Arp3 CA-binding site significantly decreased nucleation activity (Figs. 2*A* and S4). For instance, Arp3(R403D, K404D) and Arp3(W191A, T192A) showed 20% and 17% of the WT activity when activated by 1.5 μM monomeric Las17_281–633_. Mutations at the Arp2–ARPC1 CA site also decreased the activity of the complex (Figs. 2*B* and S5). Of the five Arp2–ARPC1 CA site point mutants, three mutants showed severe defects, with maximum polymerization rates (MPRs) ranging from 6 to 10% of the WT at 1.5 μM Las17_281–633_. Unexpectedly, the Arp3(R371D) point mutant, which was the most defective Arp3 site mutation in the binding assay, retained 56% of the WT activity. Together, our results show that both CA-binding sites are important for maximal activation of budding yeast Arp2/3 complex *in vitro*, consistent with results from the human Arp2/3 complex (9). Furthermore, they show that even with a strong binding defect in the Arp3 site (Arp3(R371D)), the complex can still be activated by Las17.

The Arp3–CA and Arp2–CA fusions were the most defective mutations in the pyrene actin polymerization assays (Fig. 2, *A*–*D*). Interpretation of the CA fusions in the pyrene actin polymerization assays is complicated because fusing CA to the complex has both activating and inhibitory effects. The fusions allow CA to engage each binding site, which helps stimulate the short pitch conformation (20), but because they lack a V segment, they fail to recruit actin monomers to the barbed ends of the Arps, an important step for WASP-mediated activation (15). In addition, structural data suggest that the CA fusions block interactions of the Arp subunits with the first actin subunits in the daughter filament (9, 46, 47). The inhibitory effects of the CA fusions dominate under the assay conditions we test here.

### Dimerization of Las17 increases the activity of mutants at both CA-binding sites

Previous work showed dimerization of N-WASP could partially compensate for mutations in the *Homo sapiens* Arp3 CA-binding site (9). WASP dimers were hypothesized to rescue Arp3 site defects by allowing actin monomers to be delivered to the Arp3 site without engagement of CA at that site (9). Therefore, we asked if dimerization of Las17_281–633_ increased activity of the Arp3 CA-binding site mutants. We found that the WT and all Arp3 CA-binding site mutants were more potently activated by GST-Las17_281–633_ than by the monomeric activator (Figs. 2*C* and S4). At 1.5 μM total Las17 (1.5 μM monomer or 0.75 μM dimer), the magnitude of dimerization-increased activation ranged from 1.5-fold to 3.1-fold, depending on the mutant. However, the increased activity caused by dimerization of Las17 was not limited to mutants at the Arp3 site; Arp2–ARPC1 mutants were also activated more strongly by dimerized Las17_281–633_ (Figs. 2, *D* and *E* and S5). Together, these data demonstrate that dimerization of Las17 can partially compensate for mutations in either of the two CA-binding sites in budding yeast Arp2/3 complex.

### CA-binding site mutations decrease NPF-independent Arp2/3 complex activity

To better understand the role of each of the binding sites in stimulating the short pitch conformational change, we asked whether they influenced the NPF-independent activity of Arp2/3 complex (41, 45). This activity depends on the ability of the complex to weakly populate the short pitch conformation in the absence of WASP (20, 48). We found that all CA-binding site mutations decreased the NPF-independent activity of the complex to some extent, supporting a model in which both CA-binding sites contribute to the short pitch conformational switch (Figs. 2*F* and S6) (20).

### Defects in the CA-binding site mutations have complex effects on endocytic actin assembly

We next asked how the CA-binding site mutations influenced actin assembly at endocytic sites in yeast. We and others have shown that treatment of budding yeast with the Arp2/3 complex inhibitor CK-666 results in the complete loss of endocytic actin networks (Fig. S7) (49, 50, 51). Therefore, we hypothesized that the CA-binding site mutations would decrease or eliminate actin assembly at endocytic sites. To monitor actin dynamics, we expressed mutant Arp2/3 complex subunits under their native promoter in haploid strains lacking the WT subunit. The strains expressed Las17 N-terminally tagged with mNeongreen (mNG-Las17) and TagRFP-T-labeled Abp1, an actin filament–binding protein that marks endocytic actin filaments (32). As noted previously, there are multiple yeast proteins that contain CA or A sequences, so while we reasoned that these experiments would allow us to investigate the importance of each CA-binding site, it is important to note that their design does not allow us to distinguish whether any observed phenotypes represent a loss of interaction with Las17 alone or with multiple NPFs.

**Figure 3:**
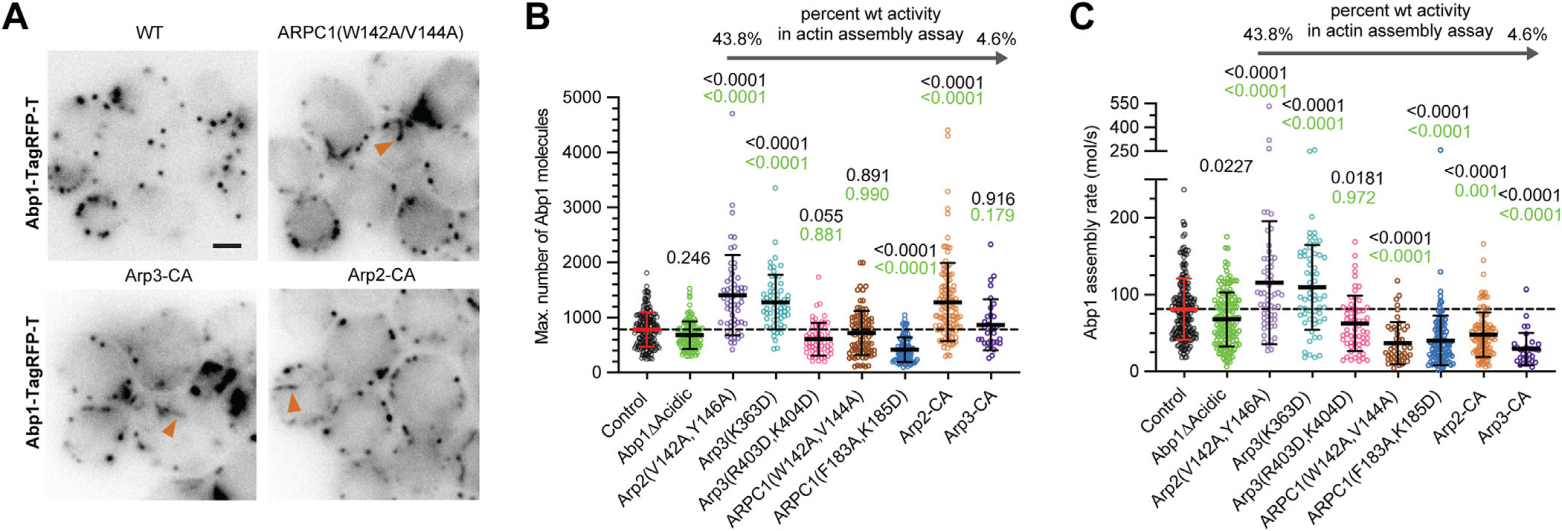
**CA-binding site mutants alter but do not block endocytic actin assembly.***A*, widefield fluorescence micrographs of budding yeast strains harboring Abp1-TagRFP-T. Brightness and contrast were adjusted individually for each image to optimize visualization of endocytic actin patches. *Orange arrowheads* mark aberrant strand-like actin structures. Scale bar represents 2 μm. *B*, plot of the maximum number of Abp1-TagRFP-T molecules at endocytic sites in mutant and control strains. Statistical significance was measured with an ordinary one-way ANOVA with indicated *p* values for comparison to control strain (*black*) and Abp1Δacidic strain (*green*) calculated with a Dunnet’s test. Error bars represent standard deviation. *Dashed horizontal black line* marks the average maximum number of molecules of Abp1-TagRFP-T in the control strain. Each data point represents a single endocytic punctum. *C*, plot of the Abp1 assembly rate for mutant and control strains. Statistical significance for plots was measured as described in *B*.

We made five point mutations in the fluorescently tagged background based on their activity in the pyrene actin polymerization assay (Fig. 2, *A*–*D*): the two most defective point mutants in the Arp3 site (K363D and R403D/R404D) and the three most defective point mutants in the Arp2–ARPC1 site (ARPC1(W142A, V144A), ARPC1(F183A, K185D), and Arp2(V142A, Y146A)). We also made the Arp2 and Arp3 CA fusions in the labeled background. Cortical puncta of Abp1-TagRFP-T were visible in each of the strains, indicating that none of the mutants completely blocked assembly of endocytic actin filaments (Fig. 3*A*, Videos S1 and S2). In some mutants, we observed aberrant actin structures that appeared as clumps or elongated cytoplasmic strands of Abp1-TagRFP-T fluorescence (Fig. 3*A*, Videos S1 and S2). The strands were connected to the cortex and only present in strains with Arp2/3 complexes that were severely defective in the pyrene actin polymerization assays.

Given their defects in Arp2/3 complex activation, we wondered if the mutant strains would assemble less actin in the patches. Because of its abundance and specificity for actin filaments at endocytic sites, Abp1 is frequently used as readout of the concentration of polymerized endocytic actin (32). However, Abp1 contains both an actin filament–binding domain and acidic segments that bind Arp2/3 complex (52), so changes in Abp1 signal within endocytic patches could result from changes in actin polymerization or defects in binding to Abp1. To better understand the role of the acidic segments, we mutated them both to GSGSG and visualized Abp1 recruitment to endocytic patches. Abp1Δacidic recruitment was slightly decreased in these strains, with an average maximum of 683 molecules recruited compared with 783 molecules of Abp1, though this ∼13% decrease was not statistically significant (Figs. 3*B* and S8). These data indicate that Abp1 recruitment to endocytic patches is primarily because of its ability to bind actin filaments, but to a lesser extent, Arp2/3 complex binding may also contribute. Consequently, we proceeded with the use of Abp1 as a marker for endocytic actin, but where appropriate, we discuss the potential caveats of this analysis.

Analysis of the cortical Abp1-TagRFP-T puncta in the Arp2/3 mutant strains revealed that while several of the CA-binding site mutations caused significant changes in the amount of Abp1 recruited, there was no clear correlation between the number of Abp1-TagRFP-T molecules recruited and the *in vitro* activity of the complex (Fig. 3*B*). For example, of the four most defective complexes in the pyrene actin polymerization assay, one mutant (Arp2–CA) assembled more Abp1 on average, one mutant (ARPC1(F183, K185D)) assembled less, and two mutants (Arp3–CA and ARPC1(W142A, V144A)) assembled nearly the same amount as the WT complex (Fig. 3*B*). While the Abp1-TagRFP-T signal may slightly underestimate the amount of polymerized actin for the reasons discussed previously, the large decrease in Abp1 in the ARPC1(F183, K185D) strain is probably not caused by reduced binding of Abp1 to Arp2/3 complex, given that the number of Abp1 molecules in the endocytic patches in the ARPC1(F183, K185D) strain is decreased dramatically (419 *versus* 783 molecules) compared with the Abp1Δacidic strain.

Despite the lack of correlation between *in vitro* Arp2/3 complex activity and the amount of actin assembled at endocytic sites, there was a correlation between the rate of Abp1 accumulation and *in vitro* branching nucleation by Arp2/3 complex (*R*^2^ = 0.62). More defective mutants showed decreased Abp1-TagRFP-T accumulation rates (Figs. 3*C*, S8 and S9*A*). The decreased assembly rates cannot be explained by defects in the Abp1–Arp2/3 complex interaction; whereas the Abp1Δacidic strain showed a slight decrease in the Abp1 assembly rate, the most nucleation-defective Arp2/3 complex mutants showed significantly lower assembly rates than the Abp1Δacidic strain (Fig. 3*C*). These data suggest that the rate of actin assembly at endocytic sites is decreased in the most defective mutants. Despite their defects in the assembly rate, some of these mutants accumulated more Abp1 than the control strain because their actin assembly phases were prolonged (Fig. S9*B*). Together, these data suggest that mutations that reduce branching nucleation activity of the complex slow actin assembly at endocytic sites, but in most CA-binding site mutants, slowed actin assembly is compensated for by an extended actin assembly phase. Surprisingly, two mutants (Arp3(K363D) and Arp2(V142A, Y146A)) assembled Abp1-TagRFP-T more rapidly than the strain expressing WT Arp2/3 complex for reasons we do not currently understand (Figs. 3*C* and S8).

### Both CA-binding sites are required for normal endocytic actin internalization

**Figure 4:**
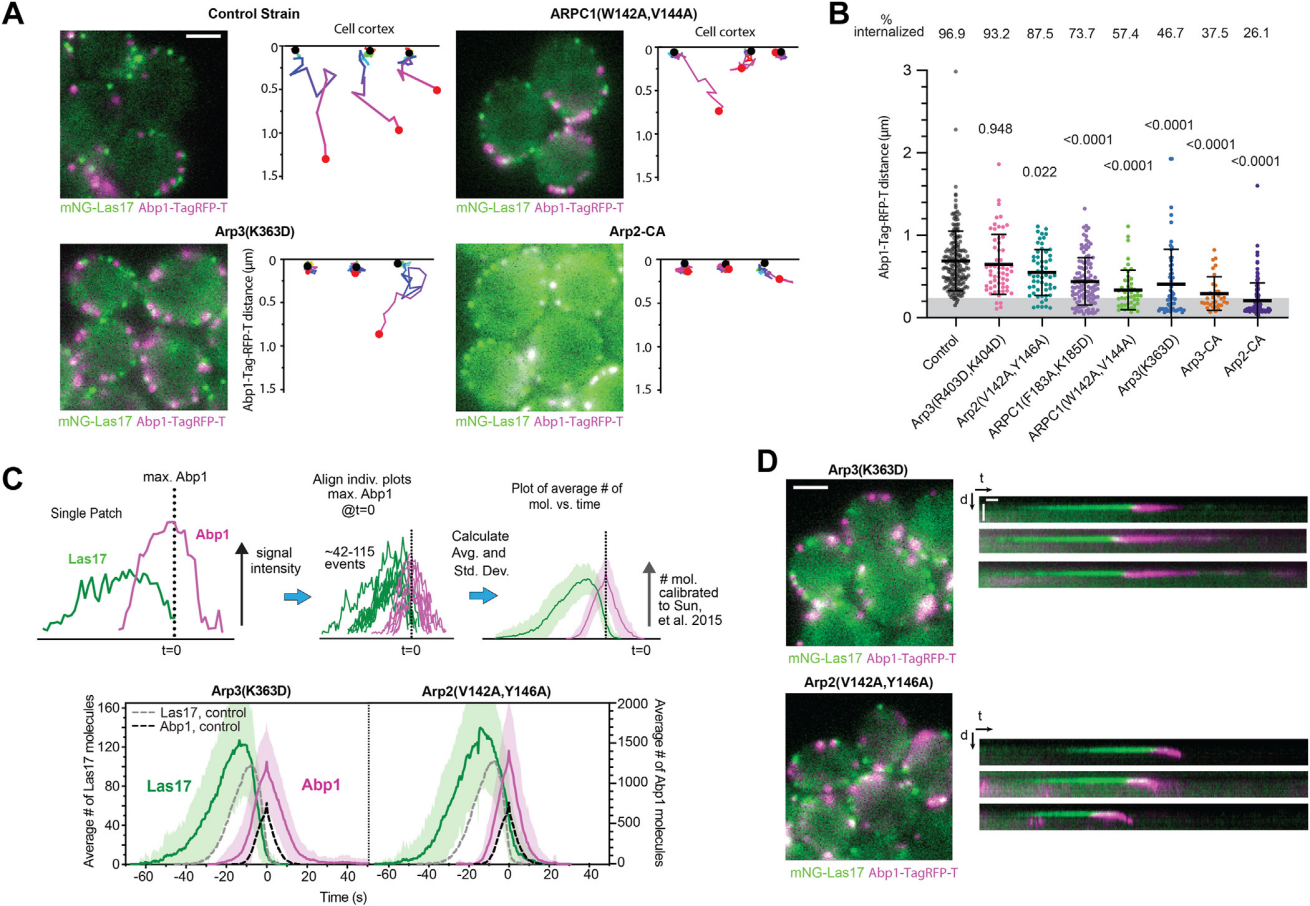
**Mutations in both Arp3 and Arp2–ARPC1 binding sites cause endocytic actin internalization defects.***A*, widefield fluorescence microscopy images of control and mutant *Saccharomyces cerevisiae* strains expressing mNG-Las17 and Abp1-TagRFP-T. Scale bar represents 2 μm. Plots of the trajectories of three representative endocytic events are shown to the *right*, based on the position of Abp1-TagRFP-T over time. The start of each trajectory is marked with a *black circle*, the end with a *red circle*, and the color gradient (*red*, *green*, *cyan*, *blue*, *violet*, and *magenta*) of the line indicates the time within the trajectory. *B*, analysis of the maximum distance traveled by Abp1-TagRFP-T puncta from their initial position on the membrane. Punta that moved more than 0.25 μm (*gray bar*) from their initial position were considered internalized. Each data point represents a single endocytic punctum. Data were collected from at least three separate videos. Error bars represent standard deviation. Statistical significance was measured with an ordinary one-way ANOVA with indicated *p* values for comparison to control strain calculated with a Dunnet’s test. *C*, schematic of procedure for generating average number of molecules *versus* time plots. Plots of the average number of mNG-Las17 or Abp1-TagRFP-T molecules *versus* time for the Arp3(K363D) and Arp2(V142A, Y146A) mutants are shown at the *bottom* of the panel. *D*, widefield fluorescence microscopy images of mutant *S*. *cerevisiae* strains expressing mNG-Las17 and Abp1-TagRFP-T. Scale bar represents 2 μm. To the *right* are kymographs of individual representative endocytic events. Scale bar for distance (d) represents 1 μm, and scale bar for time (t) represents 5 s.

To determine how the CA-binding site mutations influence actin network function, we used Abp1-TagRFP-T to measure endocytic actin internalization. We imaged the cells in the equatorial plane and measured inward movement of Abp1-TagRPF-T puncta from the membrane. Puncta that moved more than 0.25 μm from their origin were considered successfully internalized, whereas those that moved shorter distances were not (Fig. 4*A*). In control cells expressing WT Arp2/3 complex, 97% of the Abp1 puncta internalized (Fig. 4*B*). Point mutations at both CA-binding sites caused significant decreases in internalization, indicating that both sites contribute to normal endocytic actin assembly. However, none of the mutations, even those that strongly blocked NPF binding at one of the CA sites, completely blocked internalization (Fig. 4*B*). The severity of internalization defects among all mutants did not correlate well with their *in vitro* nucleation activity or the amount of Abp1-TagRFP-T accumulated at endocytic sites (Figs. 4*B* and S10). For example, although the Arp3(R403D, K404D) mutant shows the same activity or worse activity in the pyrene actin polymerization assays as the Arp3(K363D) mutant (Fig. 2), the former has no internalization defect, whereas the latter displays the strongest internalization defect among all point mutants (Fig. 4*B*). The dynamics of actin assembly also failed to predict internalization defects. For instance, the Arp3(K363D) and Arp2(V142A, Y146A) mutants are among the most and least defective mutants in internalization, respectively, but they show nearly identical dynamics (and maximum number of molecules recruited) of Abp1-TagRFP-T (Figs. 4, *B*–*D* and S8). These findings suggest that factors other than the total amount of actin or the dynamics of the actin network—for example, network architecture—play an important role in the ability of a network to produce force at endocytic sites.

### Mutants with significant internalization defects show increased Las17 at endocytic sites

**Figure 5:**
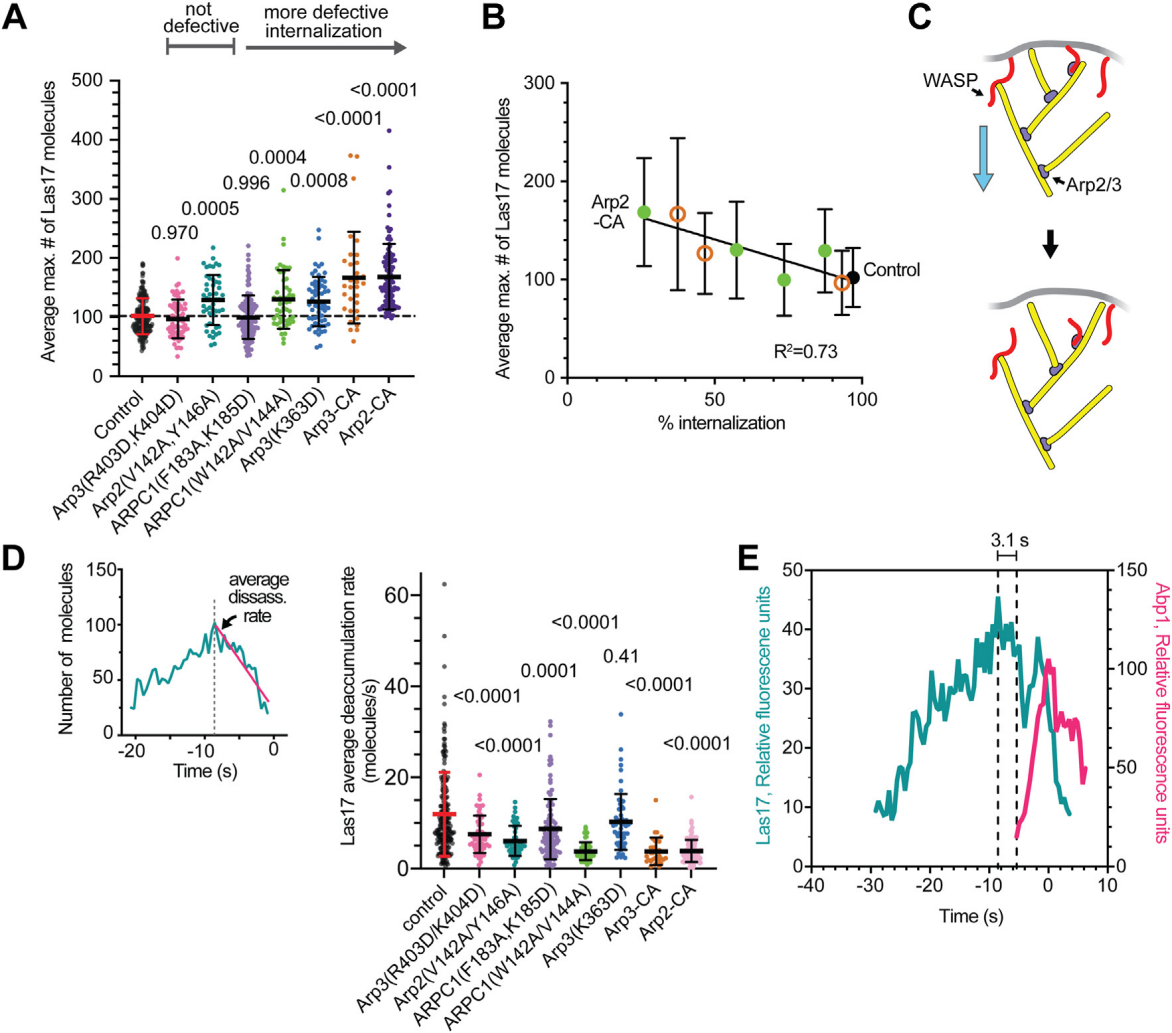
**Mutants with defective Abp1 internalization show increased Las17 concentrations and decreased Las17 deaccumulation rates.***A*, plot of the average maximum number of Las17 molecules at endocytic sites for the control and mutant strains. Mutants are ordered from *left* to *right* based on their influence on endocytic internalization. Error bars represent standard deviation. *Dashed horizontal black line* marks the average maximum number of molecules of mNG-Las17 in the control strain. Statistical significance was measured with an ordinary one-way ANOVA with indicated *p* values for comparison to control strain calculated using a Dunnet’s test. Each data point represents a single endocytic punctum. *B*, plot of the average maximum number of mNG-Las17 molecules at endocytic sites *versus* the percent internalization (from Fig. 4*B*) for control and mutant strains (*orange circles*, Arp3 mutants; *green filled circles*, Arp2-ARPC1 site mutants). *C*, diagram illustrating one possible mechanism for negative feedback between Las17 and filamentous actin (24). Las17 bound to the membrane is connected to the actin cytoskeleton *via* interaction of its CA segment with Arp2/3 complex at the side of a (unbranched) filament or its V (WH2) segment with the barbed end of an actin filament. The actin network treadmills inward and pulls Las17 off the membrane. *D*, *left*, plot of the number of mNG-Las17 molecules *versus* time for a single endocytic event. The slope of the decrease in Las17 molecules over time (*magenta line*) is used to plot the average Las17 deaccumulation rate for control and mutant strains (*right*). Statistical significance was measured as described in *A*. Error bars represent standard deviation. *E*, plot of the fluorescence intensity of mNG-Las17 and Abp1-TagRFP-T *versus* time for one endocytic event in which the signal of mNG-Las17 begins to decrease before the assembly of Abp1-TagRFP-T is initiated. *Dashed vertical lines* indicate peak of mNG-Las17 intensity and start of Abp1-TagRFP-T intensity.

While CA-binding site mutations caused variable defects in actin accumulation, their influence on Las17 was more consistent. Specifically, the mutations with decreased internalization rates caused increased accumulation of Las17 in the endocytic patches (Fig. 5, *A* and *B*). For instance, compared with the control strain, the average peak number of molecules of mNG-Las17 is ∼20% greater in internalization defective point mutants Arp3(K363D) and ARPC1(W142A, V144A) and ∼60% greater in the CA-fusion strains. These observations are consistent with a previously proposed negative feedback model in which polymerizing actin removes NPFs from endocytic sites, either directly, by dragging them off the membrane, or indirectly, by changing the curvature of the membrane (Fig. 5*C*) (24). In this model, defects in the actin network or reduction of the Arp2/3-NPF affinity—as caused by the CA-binding mutations—could reduce the rate of Las17 dissociation from the membrane, explaining its increased accumulation. Consistent with this, three of the four of the most defective mutants showed significantly decreased rates of mNG-Las17 deaccumulation (Fig. 5*D*). However, while these observations are consistent with a negative feedback model, tracking of individual puncta revealed many instances (*e.g.*, 70 of 195 events or 35.9% in the WT strain) in which mNG-Las17 puncta begin to deaccumulate before actin assembly is detected (Figs. 5*E* and S11). These findings suggest that mechanisms other than the negative feedback mechanism must also contribute to deaccumulation of mNG-Las17 from endocytic puncta.

### Mutations at both CA-binding sites cause defects in actin-based bead motility

**Figure 6:**
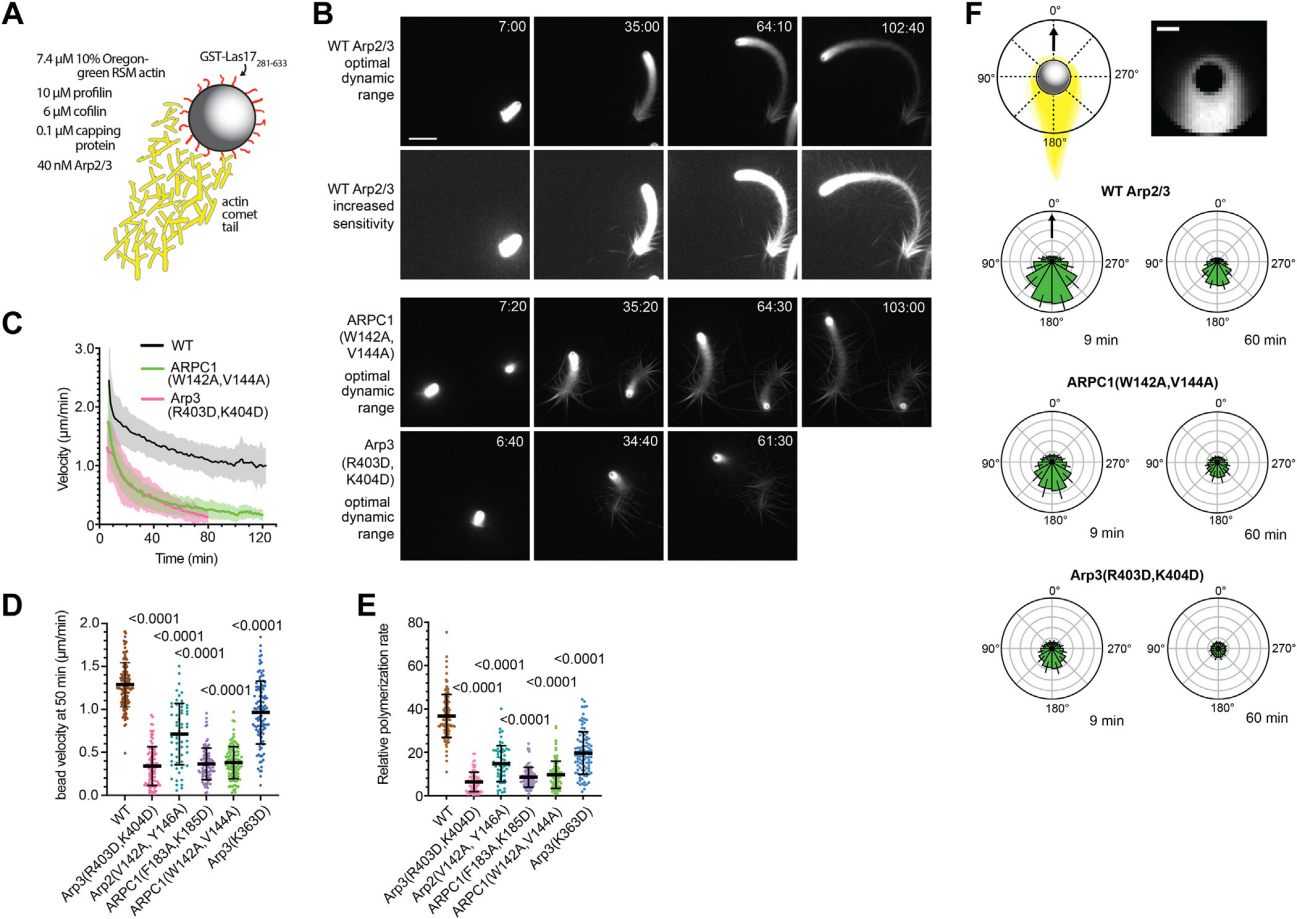
**Mutations at both CA-binding sites cause defects in actin-based bead motility.***A*, schematic of actin-based bead motility assay. *B*, widefield fluorescence microscopy images of bead motility assays using WT and mutant Arp2/3 complexes. Two of the most defective point mutants are shown. The brightness and contrast of the second row of images is adjusted to show actin filament bundles more clearly. All other images are adjusted to same brightness and contrast values. Scale bar represents 20 μm. *C*, average bead velocity *versus* reaction time for reactions with WT or the most defective point mutants. *Shaded area* shows the standard deviation (n = 63–145 beads). *D*, plot of the bead velocity at 50 min. Statistical significance was measured with an ordinary one-way ANOVA with indicated *p* values for comparison to control calculated using a Dunnet’s test. Each data point represents an individually tracked bead. *E*, plot of the relative actin polymerization rate at the surface 50 min into the motility reaction for mutant and WT complexes. The relative polymerization rate was calculated by multiplying the velocity of the bead by the intensity of actin at the bead surface (22). Statistical significance was measured with an ordinary one-way ANOVA with *p* values as described in *D*. *F*, angular intensity plots showing the average actin fluorescence intensities within an annulus centered on the bead. Scale bar represents 2 μm. Beads were aligned based on their direction of motion (*black arrow*) to calculate average fluorescence. Error bars represent standard deviation (n = 90–112).

Our data show that there is not a strong correlation between the ability of Arp2/3 complex mutants to support endocytic invagination and their activity in the pyrene actin assembly assay (Fig. S10). Therefore, to better understand how mutations at each CA-binding site could influence the function of Arp2/3 complex *in vivo*, we tested their activities in a reconstituted actin-based motility assay. This assay more closely matches physiological conditions because Las17 is clustered on the surface of a bead, mimicking the clustering of Las17 on membranes during endocytosis. Furthermore, the mix of proteins required to form the actin comet tail that drives bead motility includes a core set of actin regulatory proteins that function at endocytic sites, some of which are known to influence WASP-mediated activation of Arp2/3 complex (22, 53, 54) (Figs. 6*A* and S12*A*).

Though we previously optimized the actin-based bead motility with ScArp2/3 complex (55), reactions in PC buffer required reoptimization. Under the newly optimized conditions (Fig. 6*A*), WT Arp2/3 complex supports the formation of actin comet tails, with branched actin filaments nucleating at the surface of the bead and actin disassembling from the comet tail (Fig. 6*B*, Videos S3 and S4). The velocity of the bead is highest at the beginning of the reaction (∼2.5 μm/min), but it slows and approaches a lower steady-state velocity of 1 μm/min later in the reaction, presumably because the concentration of soluble actin monomers decreases until steady state is reached (Fig. 6*C*) (55). With brightness and contrast values adjusted to maximize sensitivity, bundled filaments can be seen protruding from the middle and back ends of the comet tail (Fig. 6*B*). These bundles remain attached to the tail as it transitions from exclusively diffuse fluorescence—presumably emitted by densely packed branched filaments—to multiple bundles arranged approximately perpendicular to the bead trajectory. Filament bundling, while not typically observed in actin-based bead motility assays (56), may be caused by the increased electrostatic shielding of the PC buffer in combination with the crowding agent, methylcellulose, which is used in the bead motility reactions to decrease Brownian motion of the beads (57, 58).

Point mutations at both the Arp3 and the Arp2–ARPC1 CA-binding sites caused significant defects in the bead motility assay. All six point mutants showed decreased bead velocity, with the ARPC1(F183A, K185D) and the Arp3(R403D, K404D) mutants causing the greatest reduction compared with the WT complex (Fig. 6, *C* and *D*, Videos S3 and S4). The relative polymerization rate of actin filaments at the bead surface was decreased in each of the point mutants, consistent with their decreased activities in the pyrene actin polymerization assays (Fig. 6*E*). Each of the point mutants also showed loss of actin asymmetry on the bead at later stages in the reaction (Fig. 6*F*), possibly because of further decreases in the rate of branch nucleation at the bead surface as actin monomers were depleted.

**Figure 7:**
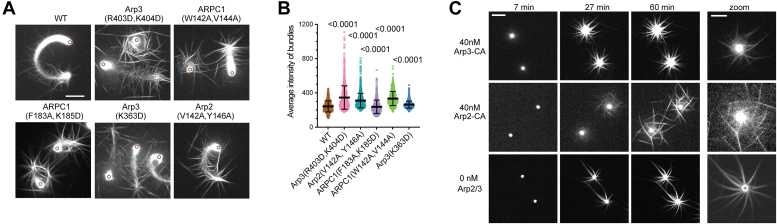
**Filament bundling in motility reactions withWTandmutantArp2/3 complexes.***A*, images of reactions with WT or mutant Arp2/3 complexes at 60 min ± 45 s adjusted at a brightness and contrast level that shows actin filament bundles clearly. *Brown circles* show location of bead. Scale bar represents 20 μm. *B*, plot of maximum fluorescence intensity of filament bundles along the trajectory of beads in WT and mutant reactions. Statistical significance was measured with an ordinary one-way ANOVA with indicated *p* values for comparison to WT control calculated using a Dunnet’s test. Note that the ARPC1(F183A, K185D) has a slightly lower but statistically significant average bundle intensity than the WT. Each data point represents the measurement of a single filament bundle around the bead. *C*, images of beads in reactions with CA-fusion Arp2/3 complexes or a reaction without Arp2/3 complex at three time points, with each image set at the same brightness/contrast settings (*left*, scale bar represents 20 μm) or zoomed-in images of each reaction at optimal time point and brightness and contrast settings to show diffuse actin networks in reactions with the CA fusion complex (*right*, scale bar represents 10 μm).

In addition to a decreased actin polymerization rate, some mutant complexes produced actin networks with increased bundling compared with WT Arp2/3 complex (Fig. 7*A*). The intensity of bundles was brighter in four of the five mutants compared with the WT (Figs. 7*B* and S12*B*). In addition, the bundles often appeared disorganized, lacking the fishbone pattern observed in the WT. In some mutants, filaments occasionally bundled in front of the bead to block motility (Fig. 7*A*, Videos S3 and S4). This “bead corralling” did not occur in the WT reactions. Previous data showed that branching nucleation antagonizes bundling (59), so WASP CA-binding site mutants may influence the actin networks both directly by reducing the branching rate and indirectly by failing to prevent bundling.

In contrast to the CA-binding site point mutants, Arp3–CA and Arp2–CA fusion mutants failed to form comet tails or support motility and instead formed asters or disconnected bundles around the bead (Fig. 7*C*, Video S5). These asters projected from or surrounded a diffuse haze of OregonGreen-actin fluorescence near the bead, which may represent regions of branched actin filaments. Filament bundles appear to form from these regions, consistent with a model in which the CA fusions nucleate actin filament networks with low-branch densities that are remodeled into bundles. Bundles projecting from the bead surface were observed in reactions lacking the Arp2/3 complex (Fig. 7*C*). These bundles likely resulted from spontaneously nucleated filaments polymerizing from the bead surface. Reactions without Arp2/3 complex lacked the diffuse fluorescence around the bead observed with Arp2–CA or Arp3–CA complexes. This absence is consistent with the interpretation that the diffuse fluorescence represents branched actin filament networks nucleated at the bead surface.

## Discussion

Here we designed mutations to disrupt binding of Las17 to each of its two binding sites on *S. cerevisiae* (Sc)Arp2/3 complex. We identified two point mutants with moderate Las17-binding defects at the Arp2–ARPC1 site and one with strong binding defects at the Arp3 site. Using *in vitro* actin polymerization assays, we found that both binding sites are required for maximal activation of the budding yeast complex, consistent with previous experiments on human Arp2/3 complex (9). Importantly, an Arp2/3 complex mutant (Arp3(R371D)) in which Las17 is strongly (or completely) blocked at the Arp3 site retained considerable Las17-stimulated branching nucleation activity. While this finding raises the possibility that Las17 might trigger branching nucleation by engaging the Arp2 site alone, it is also plausible that the residual activity of Arp3(R371D) mutant requires weak binding at the defective Arp3 CA-binding site. Therefore, whether binding to each site additively increases the activity of the complex or whether simultaneous engagement is strictly required is still an important open question.

We found that mutations at the CA-binding sites caused defects in binding both Las17 and the CA segment of Myo5. We expect that point mutations we describe here will also influence binding of the other yeast NPFs, including Abp1 and Pan1, as well as the dual activator/inhibitor of Arp2/3 complex, Crn1, which contains a CA segment (45, 52, 60). However, because Abp1 and Pan1 lack a C segment, their ability to activate Arp2/3 complex may be influenced only by the A site mutations (52, 60, 61) *S. cerevisiae* also expresses an NPF from the WDS protein family, Ldb17, which lacks C segments or A segments but is present at endocytic sites (62). WDS proteins bind to Arp2/3 complex on a surface distinct from the CA-binding sites, so we do not expect the CA-binding site mutations to directly influence Ldb17 binding (63). By activating Arp2/3 complex without a preformed actin filament, WDS proteins provide seed filaments to initiate branched actin assembly at endocytic sites (64). That we did not observe obvious reductions in the number of endocytic actin patches initiated in the mutants is consistent with a model in which the CA-binding site mutations do not significantly influence binding or activation by Ldb17.

By measuring the influence of the CA-binding sites in cells, we showed that both the Arp3 and the Arp2–ARPC1 sites are important for normal endocytic actin internalization. Therefore, while Las17 may act as an oligomer in activating the complex at endocytic sites (44, 65), the activity enhancement afforded by Las17 oligomerization is not sufficient to overcome strong defects in either the Arp3 or Arp2–ARPC1 CA-binding sites.

Previous data demonstrated that WASP dimerization could partially restore activity to Arp2/3 complex with a mutation in the Arp3 CA-binding site (9). Here, we show that dimerizing Las17 partially restored the activity of mutations at either CA-binding site. Multiple mechanisms could explain how dimerization increases the activity. For example, the avidity of the dimer might stimulate weak engagement at the defective CA-binding site, allowing a greater fraction of Arp2/3 complex to bind Las17. Dimerization could also permit delivery of an actin monomer to the defective site by the second V segment within the Las17 dimer, as proposed by Zimmet *et al.* (9) for human Arp2/3 complex. Alternatively, by facilitating simultaneous engagement at both CA-binding sites, dimerization could enhance the ability of Las17 to shift the complex toward the short pitch conformation. Titrations with at least two of the mutants, Arp3(R403D, K404D) and Arp3(K363D), saturate at lower activities than WT Arp2/3 complex (Fig. 2*C*), suggesting that avidity effects cannot fully explain the activity enhancement. However, we cannot currently determine whether actin monomer recruitment or enhanced stimulation of the short pitch conformation (or both) is responsible for activity enhancement beyond avidity effects. We note that the previous experiments with HsArp2/3 complex did not directly test whether mutations at the Arp2 site could be rescued by WASP dimerization (9), and it will be important to determine whether our observations on dimeric Las17 are generalizable, or whether there are mechanistic differences between budding yeast and human Arp2/3 complex. While several lines of evidence—including crossreactivity with WASP from different species (20)—point the similar mechanisms, other experiments have suggested there are key differences, including that the budding yeast complex populates the short pitch conformation more readily than the human complex (9, 20), and that locking the complex into the short pitch conformation bypasses the need for WASP in budding yeast but not human Arp2/3 complex (9, 20).

The impact of CA-binding site mutants on actin and Las17 dynamics at endocytic sites is consistent with the previously proposed negative feedback mechanism of Las17 removal from the membrane (24). Specifically, we found that most CA-binding site mutants showed an increase in the maximum concentration of Las17 and a decrease in the rate of Las17 disappearance from endocytic puncta. However, the negative feedback model is unlikely to be the only mechanism to remove Las17 from the membrane because, at many sites, we observed reduction in the Las17 signal before Abp1 signal was detectable. Other mechanisms are known to contribute to disassembly of the endocytic machinery, including phosphoinositide lipid modification and the actions of protein kinases (50, 66). It will be important to determine how these mechanisms influence actin disassembly and NPF dynamics at these sites.

The negative feedback mechanism explains why mutant strains accumulate filamentous actin at levels that are, in most cases, more or the same as in the control strain (24). Specifically, if the actin network removes Las17 from the membrane, defects in the network caused by the CA-binding site mutations could cause Las17 to accumulate. This could increase the concentration of filamentous actin by prolonging the actin assembly phase and/or by creating high local densities of Las17 that could drive its engagement with Arp2/3 complex. Given that Las17 contains the domains required for the barbed-end polymerase activity previously reported in WAVE1 (27), a mammalian WASP protein, it is also possible that actin accumulation is increased by the polymerase activity of excess Las17 at the membrane. While previous studies reported Arp2/3-independent actin filament nucleation by Las17 (42), this mechanism is unlikely to contribute to actin assembly in either the WT or mutant strains, as we did not find evidence of Arp2/3-independent activity by Las17 in the physiological-like buffer (Fig. S1, *I* and *J*) (67). Importantly, our data are consistent with mutational analyses in budding yeast, in which the CA (or A) segments were deleted from single or combinations of NPFs (24, 36, 37). These studies showed that NPF-mutant strains had normal or excess filamentous actin at endocytic sites. These strains, like the CA-binding site mutant strains, also showed prolonged Abp1 assembly phases and, in some cases, increased Las17 accumulation (24).

Our data suggest that factors other than the quantity and dynamics of actin filaments are important in generating functional actin networks at endocytic sites. For instance, we showed that two mutants with nearly identical concentrations and rates of accumulation of Abp1 showed very different internalization efficiencies. We also identified one mutant (ARPC1(F183A, K185D)) that assembled half the maximum amount of actin filaments on average as the control strain, yet had only a minor defect in internalization. Based on these data, we hypothesize that defects in the architecture of the actin networks constitute an important difference in their ability to internalize. Galletta *et al.* (37) also provided evidence that the architectures of endocytic actin networks are critical for their function; they showed that NPF CA segment mutations altered the ratios in endocytic actin patches of Arp2/3 complex, capping protein, and Abp1—which mark branch junctions, barbed ends, and actin filaments, respectively. Experimental evidence suggests that endocytic actin networks are densely branched and that new actin polymerizes near the membrane, pushing against the membrane to drive vesicle internalization (32, 33, 68, 69). By decreasing branching nucleation rates, mutations in the CA-binding sites may reduce branching densities in endocytic actin networks, decreasing the internalization force. Indirect effects of the mutations may also influence the architecture unproductively. For instance, decreased concentrations of activated Arp2/3 complex may decrease competition between barbed ends and prenucleation Arp2/3 complex for actin monomers, favoring elongation (70). Actin filament bundling may also unproductively influence the actin network architectures. While it is unclear if filament bundling normally occurs at the endocytic sites, several actin filament crosslinking/bundling proteins are present (34), and our bead motility assays suggested that bundling may be increased in the mutants. Bundling at endocytic sites could potentially explain the formation of the strand-like structures we observed in the most defective mutants. We anticipate that the panel of mutants we describe here will be useful for future studies investigating the mechanism of WASP-mediated activation and its role in controlling actin assembly in cells.

## Experimental procedures

### Generation of *S. cerevisiae* strains

To generate *S. cerevisiae* strains for live-cell imaging, previously generated strains harboring Arp2/3 complex mutations (strains ScBN114, 120, 147, 153, 157, 200, 315, and 323) (Table S1) were transformed with two PCR-linearized cassettes by lithium acetate transformation (10, 71). The introduction of the linearized constructs into endogenous chromosomal recombination resulted in N-terminal tagging of Las17 with mNeonGreen under a natMX6 selection marker and C-terminal tagging of Abp1 with TagRFP-T followed by kanMX6 as selection marker. Transformants were selected in YPAD media plates containing 50 μg/ml nourseothricin (clonNAT) and 200 μg/ml geneticin (G418). mNeonGreen and TagRFP-T signals were confirmed *via* live-cell imaging, and the genomic tagging was confirmed by PCR amplification of the insertion junction regions. To generate strains for purification of Arp2/3 complex, a tandem affinity His_12_-TwinStrep tag followed by a hygMX6 selection marker was introduced at the C-terminal region of the ARPC2 subunit using a modified lithium acetate procedure to generate strains ScBN399, 402, 403, 404, 405, 406, 407, 408, 411, 413, 414, and 425 (Table S1). Transformants were selected in YPAD plates containing 200 μg/ml hygromycin B. Successful tag integration was confirmed *via* PCR amplification and sequencing of the genomic ARPC2 C-terminal region. The Abp1Δacidic strain was generated by transforming a linearized cassette containing a mutated Abp1 sequence, where the N-terminal (residues 197–205) and C-terminal (residues 433–445) acidic regions were mutated to a GSGSG, followed by TagRFP-T and the kanMX6 selection marker.

### Cloning, protein expression, and protein purification

Strains expressing affinity-tagged ARPC2 were grown in YPAD media supplemented with 200 μg/ml hygromycin B to an absorbance of ∼10 at 600 nm. Subsequently, 25 g of YPD media powder and 50 mg of adenine–HCl were added per liter of culture, and growth continued to an absorbance of 20 to 25 at 600 nm. Semiwet cell pellets were then collected *via* centrifugation, resuspended in buffer W (100 mM Tris [pH 8.0], 150 mM NaCl, 1 mM EDTA, and 1 mM DTT), and stored at −80 °C. Cell pellets were thawed and resuspended in buffer W supplemented with complete protease inhibitor tablets. Cells were lysed *via* 8 to 10 passes through a microfluidizer (Microfluidics Model M-110EH-30 Microfluidizer Processor) at 23 kPSI, at which point PMSF was added to a final concentration of 1 mM. The lysate was clarified *via* centrifugation at 5000*g* for 30 min, followed by ultracentrifugation at 58,000*g* for 75 min. The clarified lysate was filtered through cheesecloth before protein precipitation with ammonium sulfate (313 g/l) for 30 min at 4 °C. The precipitate was collected *via* centrifugation at 58,000*g* for 17 min, and the pellet resuspended in buffer W. The resuspended pellet was then dialyzed extensively against buffer W. Samples were then blocked with BioLock (IBA Lifesciences) and centrifuged. The supernatant was collected and passed through a column containing Strep-Tactin-XT 4Flow affinity resin (IBA Lifesciences). The column was washed with 10 column volumes of buffer W, and protein was eluted in 1 ml fractions with buffer W supplemented with 50 mM biotin. Fractions containing the Arp2/3 complex were pooled and diluted 1:10 in Ni–NTA loading buffer (20 mM Tris [pH 8], 500 mM NaCl, and 5 mM imidazole). Diluted samples were then passed over a 5 ml Ni–NTA affinity column, washed with 10 column volumes of Ni–NTA wash buffer (20 mM Tris [pH 8], 500 mM NaCl, and 15 mM imidazole), and eluted with Ni–NTA elution buffer (20 mM Tris [pH 8], 500 mM NaCl, and 250 mM imidazole). Fractions containing Arp2/3 complex were pooled and concentrated to <5 ml using a 50 kDa molecular weight cutoff spin filter (Sartorius). As a final purification step, Arp2/3 complex samples were injected into an S200 size-exclusion column equilibrated with gel filtration buffer (20 mM Tris [pH 8] and 500 mM NaCl). Peak fractions containing pure Arp2/3 complex were pooled, buffer exchanged into 20 mM Tris (pH 8), 100 mM NaCl, 30% glycerol (v/v), flash-frozen in liquid nitrogen, and stored at −80 °C.

GST-Las17_529–633_ and Las17_529–633_ purification was performed as previously described (10). To generate the Las17_281–633_ construct, residues 281 to 633 were PCR amplified out of a parent vector containing the full-length Las17 coding sequence and inserted into a linearized pet28a+ vector with an N-terminal His_6_-GST tag *via* infusion cloning (Takara). GST-Las17_281–633_ was expressed in BL21 (DE3) RIPL cells. Overnight cultures were grown at 37 °C to an absorbance of ∼0.3 to 0.5 at 600 nm. The temperature was then reduced to 22 °C, and cultures were grown to an absorbance of 0.8 at 600 nm before the addition of 1 mM IPTG. Expression was carried out at 22 °C for 12 to 18 h. Cell pellets were harvested *via* centrifugation at 3500*g* for 20 min at 4 °C. GST-Las17_281–633_ was purified by resuspending the pellet in GST binding buffer (20 mM Tris [pH 8], 140 mM NaCl, 2 mM EDTA, and 1 mM PMSF) plus 3 complete Mini protease inhibitor tablets (Roche). Cells were lysed by sonication, and clarified cell lysate was run over a glutathione Sepharose 4B column (Cytiva), washed, and eluted with GST elution buffer (50 mM Tris [pH 8.0], 100 mM NaCl, 1 mM DTT, and 20 mM glutathione). The elution fraction was diluted 10-fold with 15 mM Tris (pH 8.0) and 1 mM DTT before injecting into a 25 ml Source30Q column pre-equilibrated with QA buffer (20 mM Tris [pH 8.0], 50 mM NaCl, and 1 mM DTT). The column was washed with QA buffer, and protein was eluted over a gradient from QA to 70% QB buffer (same as QA but containing 500 mM NaCl). GST-Las17_281–633_ was pooled, diluted in 20 mM Tris (pH 8.0), and 1 mM DTT to bring the NaCl concentration to 50 mM, then concentrated, and flash frozen.

Monomeric Las17_281–633_ was purified on Ni–NTA affinity and a cation exchange column as follows. Clarified lysate was passed over a Ni–NTA column and eluted with Ni–NTA elution buffer. Fractions were pooled, buffer exchanged into 20 mM Tris (pH 8), 100 mM NaCl, 50 mM imidazole, 0.5 mM EDTA, 1 mM DTT, followed by the addition of 3 mg tobacco etch virus (TEV) protease and incubation at 4 °C for 12 to 18 h. Cleaved protein was reloaded onto the Ni–NTA affinity column to remove His_6_-GST tag and any nondigested product. The flow-through was collected and concentrated to <5 ml using a 10 kDa molecular weight cutoff spin filter. The concentrated sample was diluted with SA buffer (50 mM Hepes [pH 7], 50 mM NaCl, and 1 mM DTT) and injected into a 6 ml Resource S cation exchange column. The column was then washed, and Las17_281–633_ was eluted using a linear gradient from 0 to 30% SB buffer (50 mM Hepes [pH 7], 500 mM NaCl, and 1 mM DTT). Pure fractions were pooled, concentrated, flash frozen, and stored at −80 °C.

LZ-Las17_281–633_ and LZ-Myo5_1146–1220_ were constructed by inserting a cysteine residue in front of the leucine zipper domain of *S. cerevisiae* Gcn4 (residues 250–281) and a 12 residue Gly-Ser linker between the TEV protease site and the N terminus of either Las17_281–633_ or LZ-Myo5_1146–1220_ in pGV67. GST-TEV constructs were overexpressed in BL21(DE3)-RIPL *E. coli* and purified using a glutathione Sepharose column (Cytiva). Eluted proteins were digested with TEV protease during dialysis. Cleaved proteins were run over a glutathione Sepharose column to remove the GST tag and any undigested protein. LZ-Las17_281–633_ was injected into a 6 ml Source15S column, washed with SA buffer (20 mM Pipes [pH 6.8], 50 mM NaCl, and 0.5 mM Tris(2-carboxyethyl)phosphine [TCEP]), and eluted over a linear gradient of 50 to 500 mM NaCl. LZ-Myo5_1146–1220_ was injected into a 6 ml Source30Q column, washed with QA buffer (20 mM Tris–HCl [8.0], 50 mM NaCl, and 0.5 mM TCEP), and eluted over a linear gradient of 50 to 500 mM NaCl. Fractions containing pure LZ-Las17_281–633_ or LZ-Myo5_1146–1220_ protein were pooled, concentrated, and incubated with 10 mM TCEP at room temperature for 30 min. After desalting, protein concentration was determined by measuring its absorbance (LZ-Las17_281–633_, ε_280_ = 9970 M^−1^ cm^−1^; LZ-Myo5_1146–1220_, ε_280_ = 6990 M^−1^ cm^−1^). Before flash freezing, LZ-Las17_281–633_ and LZ-Myo5_1146–1220_ were biotinylated using EZ-Link Iodoacetyl-LC-Biotin (ThermoScientific) following the manufacturer guidelines, resulting in more than 90% labeled protein measured by a HABA assay (ThermoScientific).

*Mus musculus* capping protein, human cofilin, and human profilin 1 were expressed in *E. coli* cells and purified as described by Narvaez-Ortiz *et al.* (55). Actin was purified from rabbit skeletal muscle (Pel Freeze; catalog no.: 41995) and labeled with pyrene iodoacetamide or 488 OregonGreen maleimide as previously described (48).

### Actin polymerization time courses

Pyrene actin polymerization assays were run under two different sets of buffer conditions. The first system, hereinafter referred to as PC buffer, mimics the physiological intracellular ionic concentration found in *S. cerevisiae* cells by elemental composition analysis (39). The physiological-like buffer (PC buffer) used in this study included three modifications compared with the study by van Eunen *et al.* The phosphate concentration was reduced from 50 to 0.001 mM, glutamate was not used as counterion, and calcium was not included in the buffer. A 5× stock of PC buffer was prepared by creating a solution with 5 μM K_2_HPO_4_, 15 mM MgSO_4_, 100 mM NaOH, 1.375 M KOH, 600 mM Pipes (from Pipes acid), and adjusting the pH to 7.0 with acetic acid. The second buffer used was KMEI (10 mM imidazole [pH 7.0], 50 mM KCl, 1 mM EGTA, 1 mM MgCl_2_, 200 μM ATP, and 1 mM DTT), a buffer commonly used in pyrene actin polymerization assays.

Reactions in PC buffer were initiated by mixing a solution of Arp2/3 complex, Las17_281–633_, ATP, and DTT in 1.3× PC buffer with 15% pyrene-labeled actin in G buffer (5 mM Tris [pH 8.0], 0.2 mM CaCl_2_, 0.2 mM ATP, and 0.5 mM DTT) to make the solution 1× in PC buffer with a final concentration of 3 μM actin, 0.2 mM ATP, and 0.5 mM DTT. Magnesium chloride and EGTA were added to the actin solution just before the reaction was initiated to make it 0.5 mM in MgCl_2_ and 2 mM EGTA. The fluorescence was measured at 25 °C in a cuvette (to avoid interference from potential light scattering) using an ISS PC1 Photon counting Spectrofluorometer with excitation and emission wavelengths set at 365 and 407 nm, respectively. Pyrene actin assays performed in KMEI buffer were set up as previously described (55). The concentration range of monomeric Las17_281–633_ tested was 75 to 2500 nM. For reactions with GST-Las17_281–633_, 10 to 3500 nM of the activator was included (those are concentrations of the GST-Las17_281__–__633_ monomer). Assays using higher concentrations of monomeric or dimeric Las17_281–633_ resulted in inhibition of actin polymerization.

The MPR of pyrene actin polymerization assays was determined by measuring the slope of each curve at each time point and converting from RFU s^−1^ to actin nM s^−1^. Data from the MPR *versus* Las17_281–633_ titration graphs were fit the following equation:

$$Y\;=\;\frac{Y^{\max}\;\times \;\left[Las17\right]}{\left(K_{1/2}\;+\;\left[Las17\right]\right)}\;+\;Y^{o}$$

where *Y* = MPR in nM s^−1^, *Y*^max^ = maximum MPR, [Las17] = the concentration of monomeric or dimeric Las17_281–633_ in nM, K_1/2_ = the [Las17_281–633_] needed to get half-maximum MPR, and Y^o^ = the MPR in the absence of Las17_281–633_. The following equation was used to calculate the percent of activity of each mutant *versus* the WT Arp2/3 complex:

$$\%\;activity\;=\;\frac{{MPR}_{mutant}^{i}\;-\;{MPR}_{actin}}{{MPR}_{WT}^{i}\;-\;{MPR}_{actin}}\;\times \;100$$

where MPR^i^_WT_ is the maximum actin polymerization rate of the WT complex at an [*i*] concentration of Las17_281–633_, MPR^i^_mutant_ is the maximum actin polymerization rate of the CA-binding mutant complex at the same concentration of Las17_281–633_, and MPR_actin_ is the MPR for 3 μM 15% pyrene-labeled RMS actin.

### Supernatant depletion–binding assays

Supernatant depletion–binding assays were performed using the biotinylated leucine zipper dimeric form of Las17_281–633_ or Myo5_1146–1220_ as follows: pure proteome magnetic streptavidin beads (Millipore) were incubated with 20 μM biotinylated LZ-Las17_281–633_ or LZ-Myo5_1146–1220_ (LZ-Myo5–CA) in TBST buffer (20 mM Tris [pH 7.5], 150 mM NaCl, and 0.1% Tween-20) for 1 h at room temperature with gentle mixing. After separation using a magnetic stand, the unbound fraction was run on an SDS-PAGE gel to determine the concentration of unbound protein. Coated beads were washed three times with TBST and three times with 1× PC buffer plus 0.05% Tween-20 to remove the unbound protein.

To determine the time required to reach equilibrium, the coated beads were diluted in 1× PC buffer plus 0.05% Tween-20 to a single concentration of LZ-Las17_281–633_ (0.74 μM) or LZ-Myo5–CA (0.96 μM). The beads were then incubated with 50 nM WT Arp2/3 complex with gentle agitation at room temperature. The reaction was stopped at time points, ranging from 5 to 120 min by magnetically pelleting the beads and quickly removing the supernatant. Each supernatant was spotted on nitrocellulose membranes and blotted using anti-Arp3 antibodies (Santa Cruz; catalog no.: sc-376625, 1:1000 dilution).

To generate the binding isotherm curve for WT Arp2/3 complex, LZ-Las17_281–633_ coated beads were diluted in 1× PC buffer plus 0.05% Tween-20 to yield a range of concentrations of the dimer from 16 nM to 2.06 μM for (LZ-Las17_281–633_)_2_ or from 25 nM to 1.6 μM for (LZ-Myo5–CA)_2_. Arp2/3 complex was then added to the samples to yield a final concentration of 50 nM. Samples were incubated with gentle agitation for 70 min at room temperature, pelleted, and the supernatant was removed for analysis. Fraction-bound data were fit to the following equation in GraphPad Prism (version 10.0.1) to determine the *K*_*D*_ value for the WT complex:

$$\frac{\left[LR\right]}{\left[R\right]}\;=\;\frac{\left[L\right]\;+\;\left[R\right]\;+\;K_{D}\;-\;\sqrt{{\left(\;-\;\left[L\right]\;-\;\left[R\right]\;-\;K_{D}\right)}^{2}\;-\;4\left[L\right]\left[R\right]}}{2\left[R\right]}$$

where *L* is the total concentration of LZ-Las17_281–633_ or LZ-Myo5–CA and R is the total concentration of Arp2/3 complex.

For supernatant depletion experiments with mutant Arp2/3 complexes, a single concentration of (LZ-Las17_281–633_)_2_ (0.6 μM) or (LZ-Myo5–CA)_2_ (0.4 μM) on the beads was used. Beads were suspended in 1× PC buffer plus 0.05% Tween-20 containing 50 nM Arp2/3 complex (WT or mutant) and incubated for 70 min at room temperature with gentle agitation. Supernatants were spotted over nitrocellulose membranes, dried for at least an hour, blocked, and blotted with mouse anti-Arp3 (Santa Cruz; catalog no.: sc-376625, 1:1000 dilution) followed by donkey antimouse IRDye 680RD (LICOR; 1:10,000 dilution). Experiments were conducted in triplicate. Concentrations were determined using a calibration curve generated by standard samples included on each membrane. Signal intensity of the dots was quantified using Image Studio Lite software (LICOR). The fraction of Arp2/3 complex bound to the LZ-Las17_281–633_/LZ-Myo5–CA-coated beads was calculated using the following formula:

$$F_{b}=\frac{\theta^{i}}{\theta^{o}}$$

where *F*_*b*_ is the fraction of Arp2/3 complex bound, θ^i^ is the total concentration of Arp2/3 minus the concentration in the supernatant after incubation with the resin charged with an [*i*] concentration of LZ-Las17_281–633_ or LZ-Myo5–CA, and θ^0^ is the total concentration of Arp2/3.

### *S. cerevisiae* growth, live-cell imaging, and data analysis

*S. cerevisiae* strains were prepared for imaging and imaged as previously described (46). Samples were imaged on a DeltaVision Ultra High-Resolution Widefield Fluorescence microscope (Cytiva) equipped with a pco.edge 4.2 sCMOS camera, an Olympus 100× 1.4 numerical aperture Oil UPlanSApo objective and B-G-O-FR polychroics. Images were collected for 1 min and 15 s using a 100 ms exposure and 10% transmission per channel. All strains were prepared and imaged in triplicate. Endocytic patch data analysis was performed using a mixture of open-source software and custom python scripts. Briefly, images were preprocessed using FIJI to correct for uneven illumination, photobleaching (72), and cytosolic signal subtraction. Las17 and Abp1 patch dynamics were analyzed using the TrackMate plugin in FIJI (73). Patches were detected in TrackMate using the following criteria: the blob estimated diameter was set to 0.5 μm, threshold to 5, and median filter was selected only for the Las17 channel. The linking maximum distance, the gap-closing distance, and the gap-closing maximum frame gap were adjusted to 0.5 μm, 0.5 μm and 2, respectively. A subset of TrackMate-selected patches was manually selected for analysis based on the following criteria: (1) the entire lifetime of the patch is recorded in the video, (2) the patch is well separated from other patches, and (3) the patch originates on the equatorial cortex. Curated tracking data were exported as .csv files and fed into a custom python data analysis script to extract trajectories from the TrackMate files. The custom python script aligned the trajectories based on the maximum fluorescence intensity of Abp1 (https://github.com/adamfries/NolenLab/blob/main/tirf_patch_analysis_v4.py). To convert fluorescence to number of molecules, corrected fluorescence values were multiplied by the ratio of the average maximum number of molecules of Abp1 and Las17 in patches reported by Sun *et al.* (74) to the average maximum corrected fluorescence signal of Abp1 and Las17 in patches in the control strain. Accumulation and deaccumulation rates were determined from the individual trajectories using the slope of all data points between first appearance and maximum value (accumulation) or all data points between the maximum and the last recorded frame with measurable intensity (deaccumulation). The distance from origin of Abp1 puncta was determined using the *x* and *y* coordinates from the processed TrackMate trajectories. Abp1 assembly times were calculated by subtracting the time of first appearance of Abp1 from the time of its maximum concentration. In all cases, images shown in figures are unprocessed except for adjustment of brightness and contrast values.

For experiments with CK-666, budding yeast cells were grown at 30 °C in YPAD medium and kept in log phase for 48 h. Before imaging, cells were harvested by gentle centrifugation, rinsed three times with CMS media, and resuspended in 50 to 100 μl of CMS media containing 2 mM CK-666 added from a 100 mM stock in dimethyl sulfoxide. Cells were immediately placed onto gelatin pads on a glass slide, covered by a coverslip and sealed with VALAP. Control cells were resuspended in CMS media plus 2% dimethyl sulfoxide. Samples were imaged on a DeltaVision Ultra High-Resolution Widefield Fluorescence microscope (Cytiva) using the parameters described previously.

### Actin-based motility assays

Polymer-based magnetic carboxylated microspheres (ProMag 3 μm; Polysciences) were activated in 20 mM Mes [pH 5.5], before incubating with 4 μM GST-Las17_281–633_ in coupling buffer (100 mM phosphate buffer [pH 7], 150 mM NaCl) overnight at 4 °C under gently rotation. Beads were magnetically pelleted, and the supernatant was removed for analysis by SDS-PAGE gel to determine the amount of GST-Las17_281–633_ bound. Functionalized beads were washed with 0.5× PC buffer, stored in 0.5× PC buffer plus 1 mg/ml bovine serum albumin, kept at 4 °C, and used for up to 3 days. Microscope slides and coverslips were salinized using chlorotrimethylsilane as previously described (75). The motility medium contained 1× PC buffer, 15 mM TCEP, 1 mM ATP, 1.5 mM DABCO, 1 mM EGTA, 0.25% methylcellulose (1500cP), 0.5 mg/ml bovine serum albumin, 40 nM Arp2/3 complex (WT or mutants), 100 nM capping protein, 6 μM cofilin, 10 μM profiling, and 7.4 μM OregonGreen 488 actin (10% labeled). GST-Las17_281–633_-coated beads were diluted 1:20 in motility medium, and 1.5 μl of the reaction mixture was placed between a salinized glass slide and coverslip. After sealing with VALAP, the reaction was monitored using a DeltaVision widefield fluorescence-inverted microscope equipped with a motorized stage, a UPlanSApo 20×/0.75 numerical aperture (Olympus) objective and a pco.edge 4.2 sCMOS camera. Images were acquired at 70 s intervals over 1 to 2 h by differential interference contrast imaging (10 ms exposure, 2% transmission) and by fluorescence excitation with a 488 nm LED (100 ms exposure, 5% transmission). For the images shown in the figures, only the fluorescence channel is shown, and the beads, when shown, were manually drawn using the differential interference contrast imaging channel as a reference.

All beads in each field of view were used in the analysis unless (a) a bead track crossed over another bead, (b) the bead diameter was larger than 3.8 μm, or (c) the complete bead trajectory was not within the field of view. The speed of the beads was calculated using the Spots module of Imaris 10.0.1 software (Bitplane). The relative polymerization rate at the bead surface was calculated as previously described (22). Specifically, the velocity of the bead was multiplied by the maximum fluorescence intensity of actin (which always occurred at the rear of the bead at the surface). We used a custom python script to determine the maximum fluorescence signal of actin (https://github.com/adamfries/NolenLab/blob/main/af_actin_lineprofile_time.ipynb). Using the Imaris-tracked bead positions and the raw microscope movies, the script analyzes the intensity line profile of the entire track at every time point and measures the local maximum fluorescence intensities (corresponding the leading and trailing edge of the bead) and the subsequent local minimum between the two (corresponding to the location of the center of the bead). Intensity values, peak to peak distances, and peak to valley distances were then recorded for all time points.

The angular intensity distribution plots were generated by a custom python script (https://github.com/adamfries/NolenLab/blob/main/af_actin_polarization_v2.ipynb). For a user-defined range of time points, the script uses the Imaris-tracked bead positions and the two-channel microscopy videos to measure fluorescence intensity within annuli with inner and outer radii of 4 and 15 pixels at each bead position (to exclude the bead and only include the fluorescence immediately around it). The polar distribution of the mean and standard deviation of the fluorescence intensity was measured with 30° binning.

The intensity bundling plots were generated using analysis performed by a custom python script (https://github.com/adamfries/NolenLab/blob/main/actin_bundling.ipynb). Using hand-annotated intensity line profiles adjacent to the established comet tails using ImageJ, the script identifies local peaks along the profile depending on prominence, width, minimum distance between peaks, and the window with which to evaluate the prominence. The number of peaks, peak intensity, and peak location along the profile were recorded.

## Data availability

Custom scripts used for data analysis can be found at the links indicated in the Experimental procedures section.

## Supporting information

This article contains supporting information (10, 74).

## Conflict of interest

The authors declare that they have no conflicts of interest with the contents of this article.
