# Supplementary material for: Both Las17-binding sites on Arp2/3 complex are important for branching nucleation and assembly of functional endocytic actin networks in S. cerevisiae
Source: J Biol Chem. 2024 Feb 16;300(3):105766. doi: 10.1016/j.jbc.2024.105766 (PMC10944109; doi:10.1016/j.jbc.2024.105766)
Supplement: Supporting Information [file mmc6.docx]

**
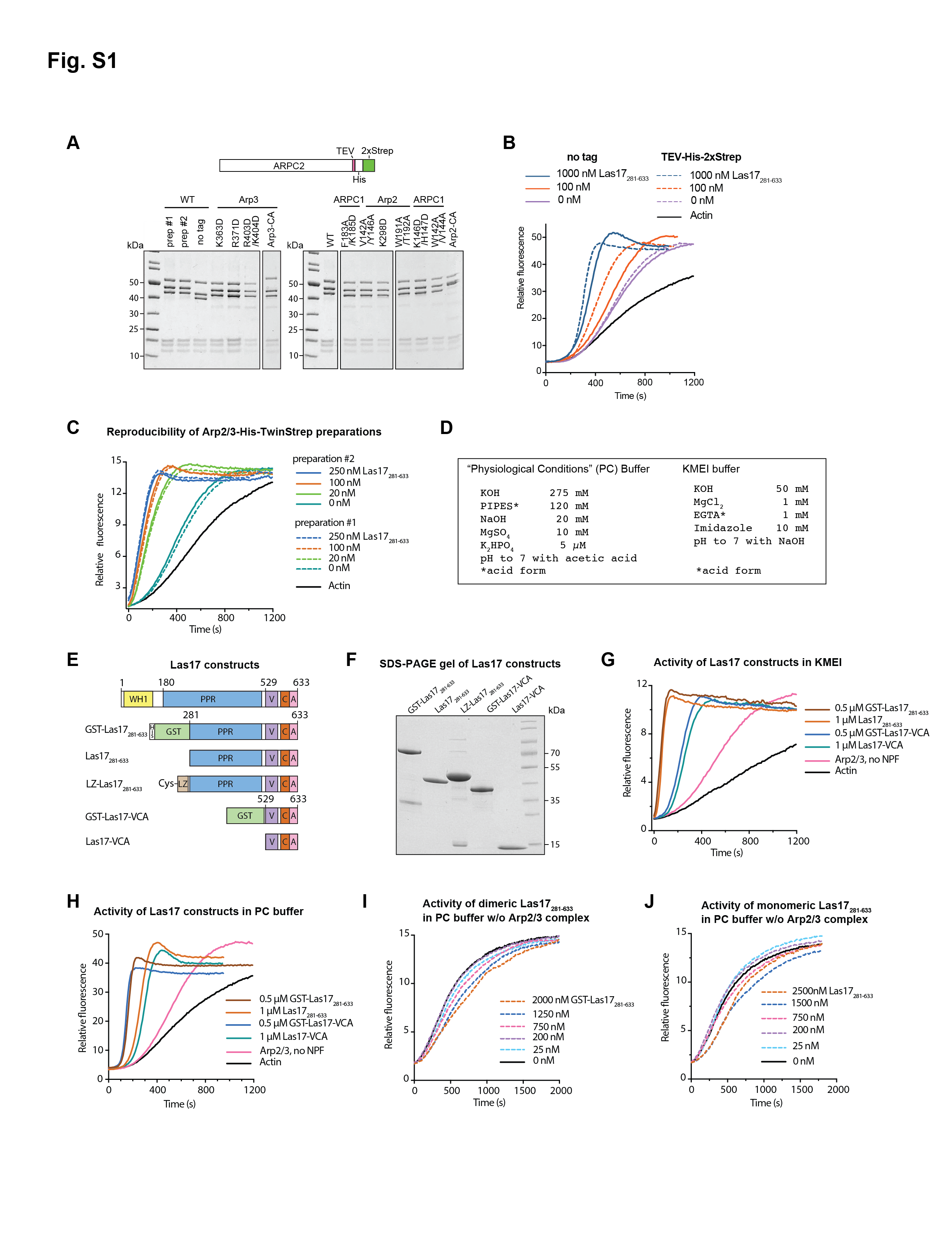
**

**Figure S1: Preparation of Arp2/3 complex and Las17 for biochemical assays.**

**A.** SDS-page gels of purified wild type and mutant Arp2/3 complexes. All complexes except for the “no tag” sample harbor the TEV-His-2xStrep tag on the C-terminus of ARPC2. **B.** Pyrene actin polymerization assays comparing the activity of the wild type Arp2/3-12xHis-TwinStrep-tagged complex to untagged wild type Arp2/3 complex. Reactions are run in PC buffer and contain 3 μM 15 % pyrene labeled actin, 20 nM Arp2/3 complex, and the indicated concentrations of Las17_281-633_. **C.** Pyrene actin polymerization assays showing the nucleation activity of 3 nM Arp2/3-12xHis-TwinStrep tagged wild -type complex from two different preparations. Reactions performed in absence or in presence of different concentrations of Las17_281-633_ showed a minimal variability in activity from batch-to-batch protein preparation. **D.** Composition of physiological conditions (PC) and KMEI buffer. **E.** Schematic diagram depicting Las17 domain organization (top) along with the different constructs used in this study. N-terminal WASP homology 1 domain (WH1), polyproline region (PPR), Verprolin-like region (V), and the central (C) and acidic regions (A). **F.** Coomassie-stained SDS-PAGE gel of purified Las17 constructs depicted in B. **G.** Time courses of pyrene actin polymerization for reactions in KMEI buffer containing 3 nM Arp2/3 wild-type complex, 3 μM (15% pyrene-labeled) rabbit skeletal muscle actin with or without different constructs of Las17, as indicated. In KMEI buffer, longer Las17 constructs activate Arp2/3 complex better than Las17 constructs that only include the VCA segment. Note that the dimer concentrations of GST-Las17_281-633_ are given, so 0.5 μM GST-Las17_281-633_ has the same number of Las17 molecules as 1 μM Las17_281-633_. **H.** Time courses of pyrene actin polymerization for reactions in PC buffer containing 20 nM Arp2/3 wild-type complex, 3 μM (15% pyrene-labeled) rabbit skeletal muscle actin with or without constructs of Las17, as indicated. Under physiological-like salt conditions, there are no major differences in Arp2/3 complex activation between long and short Las17 constructs. **I-J.** Time courses of pyrene actin polymerization for reactions containing monomeric (F) and dimeric (G) Las17_281-633_ in PC buffer with 3 μM (15% pyrene-labeled) rabbit skeletal muscle actin and no Arp2/3 complex.


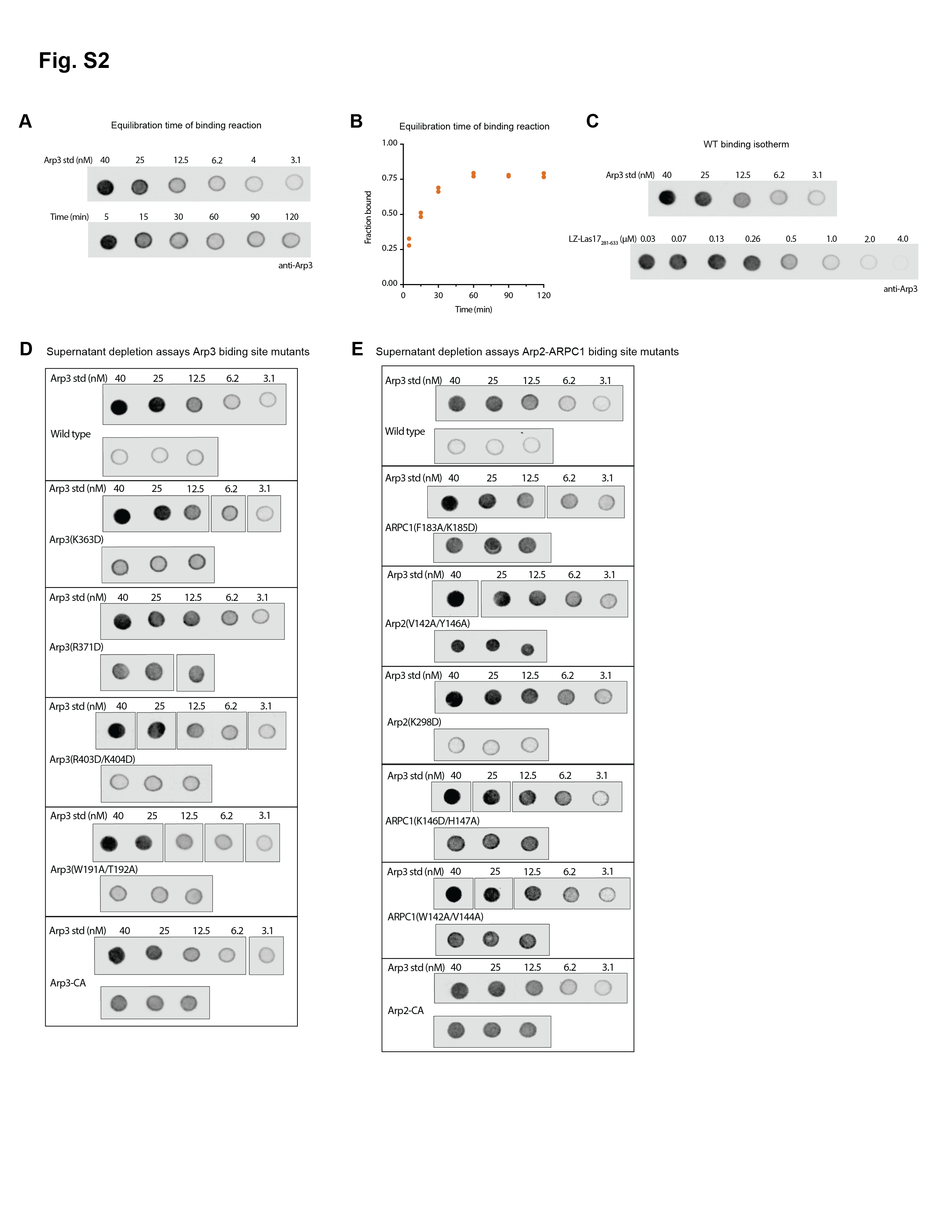


**Figure S2: Quantification of supernatant depletion binding assay. A.** Representative anti-Arp3 dot blot of a supernatant depletion binding experiment to determine the equilibration time for the binding reaction of Arp2/3 complex and Las17_281-633_. **B.** Fraction of Arp2/3 complex bound to Las17_281-633_ versus incubation time determined from the assays described in A. **C.** Anti-Arp3 dot blots of reactions containing 50 nM wild type Arp2/3 complex, and a range of concentrations of biotinylated LZ-Las17_281-633_ on the beads, as indicated. Experiments were performed in triplicate and used to generate the binding isotherm curve shown in Fig. 1E. **D-E.** Representative dot blots from binding assays using a single concentration (1.2 µM) of biotinylated LZ-Las17_281-633_ to pull down wild type, Arp3 binding-site (D), or Arp2/ARPC1 binding-site (E) mutant Arp2/3 complexes. Experiments were run in triplicate. Some blots were computationally spliced within the same membrane to align data, as indicated by spaced boxes.


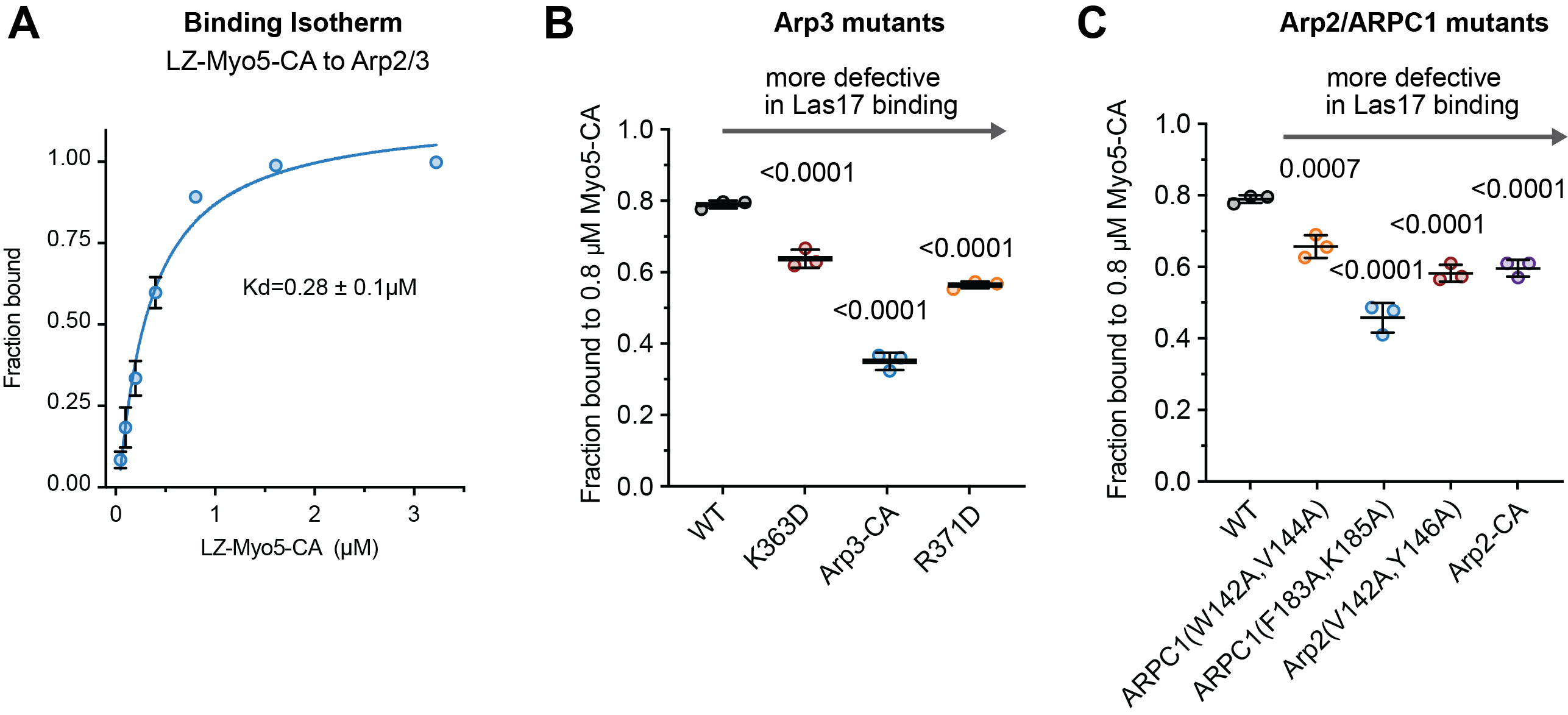


**Figure S3: Supernatant depletion binding assay using LZ-Myo5-CA. A.** Binding isotherm for LZ-Myo5_1146-1220_ generated using the supernatant depletion assay in PC buffer. Reaction was run in triplicate. Concentration on x-axis is the concentration of the LZ-Myo5-CA dimer. Error bars: standard deviation. Error bars for last three data points were approximately the same size as the data points, so were omitted. **B,C.** Plot of the fraction of wild-type and mutant Arp2/3 complex bound in supernatant depletion reactions containing 0.8 μM LZ-Myo5-CA. Mutants are ordered from left to right based on their defect on Las17 binding. Statistical significance was measured with an ordinary one-way ANOVA with p values for comparison to the wild type complex calculated using a Dunnett’s test.


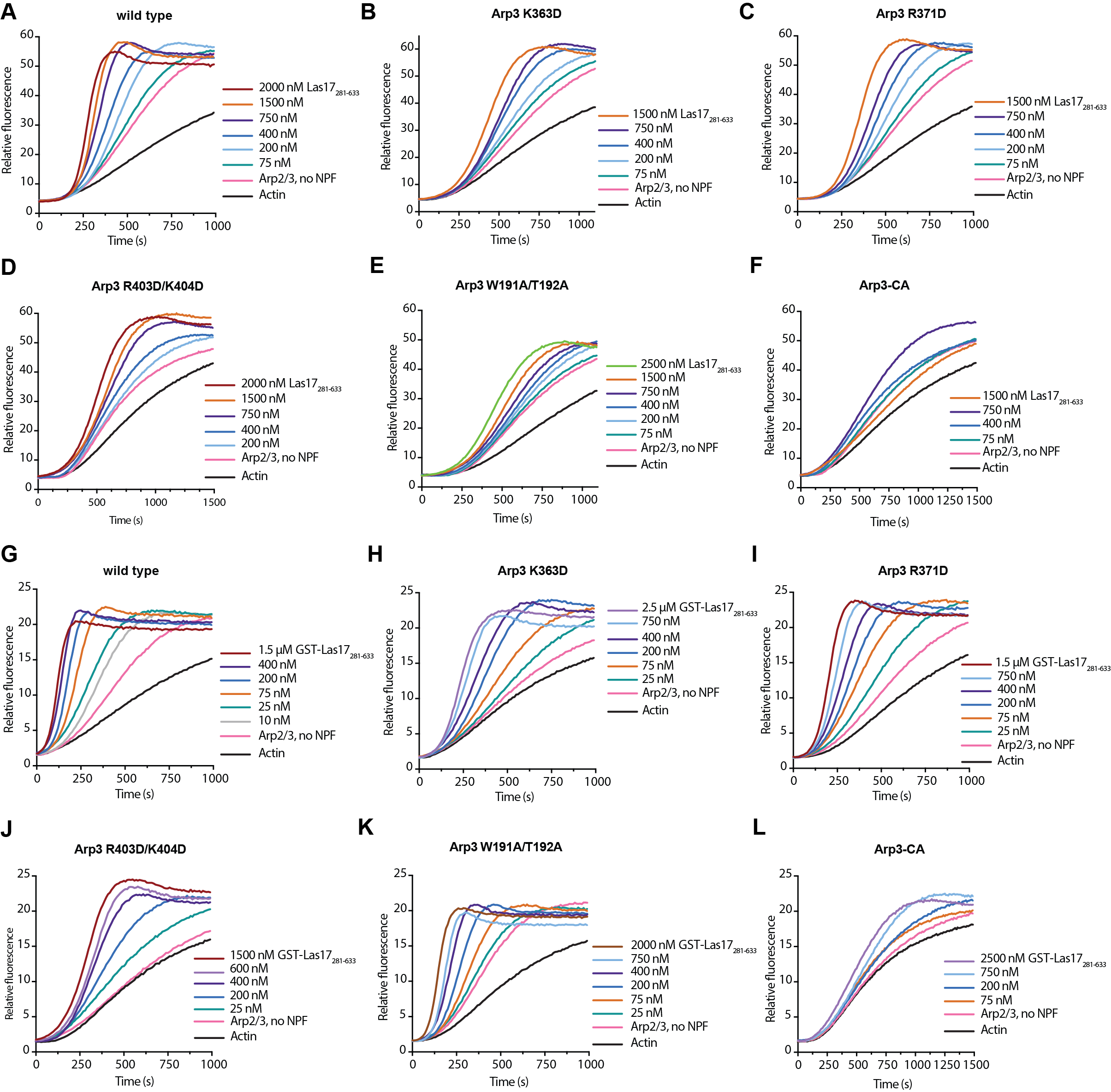


**Figure S4: Pyrene actin polymerization assays to measure the activity of the Arp3 CA-binding site mutants. A-F.** Time courses of 3 μM 15% pyrene actin polymerization in the presence of 20 nM WT Arp2/3 complex or Arp3 CA binding site mutants and the indicated concentrations of monomeric Las17_281-633_. **G-L** Same as (A-F), except dimeric Las17_281-633_ (GST- Las17_281-633_) was used. All reactions were performed in PC buffer.


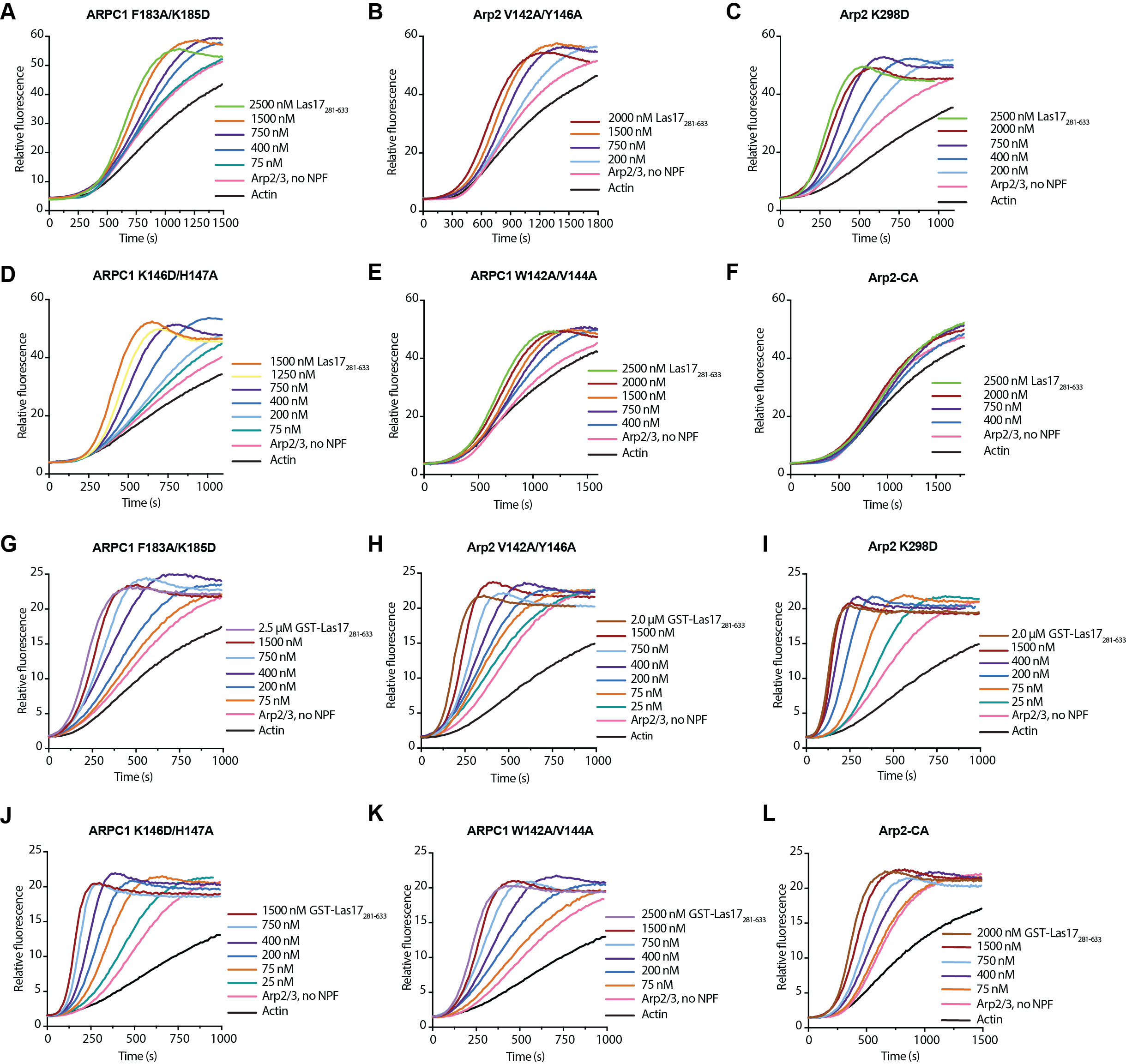


**Figure S5: Pyrene actin polymerization assays to measure the activity of the Arp2/ARPC1 CA-binding site mutants. A-F.** Time courses of 3 μM 15% pyrene actin polymerization in the presence of 20 nM Arp2-ARPC1 binding site mutants, and the indicated concentrations of monomeric Las17_281-633_. **G-L** Same as (A-F), except dimeric Las17_281-633_ (GST- Las17_281-633_) was used.

**
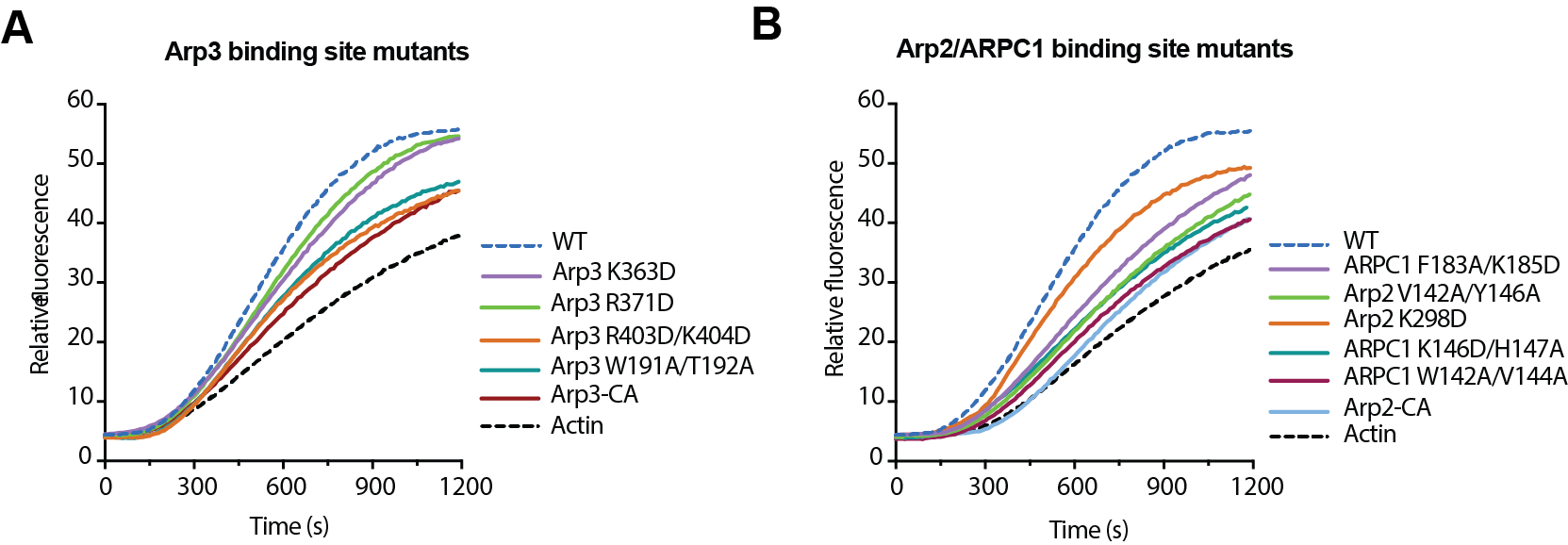
**

**Figure S6: NPF independent activity of CA mutants**. **A.** Polymerization of 3 µM 15% pyrene actin in PC buffer in the presence of 20 nM WT Arp2/3 complex or Arp3 binding site mutants. **A.** Same as A but using 20 nM WT Arp2/3 complex or Arp2/ARPC1 binding site mutants.

**
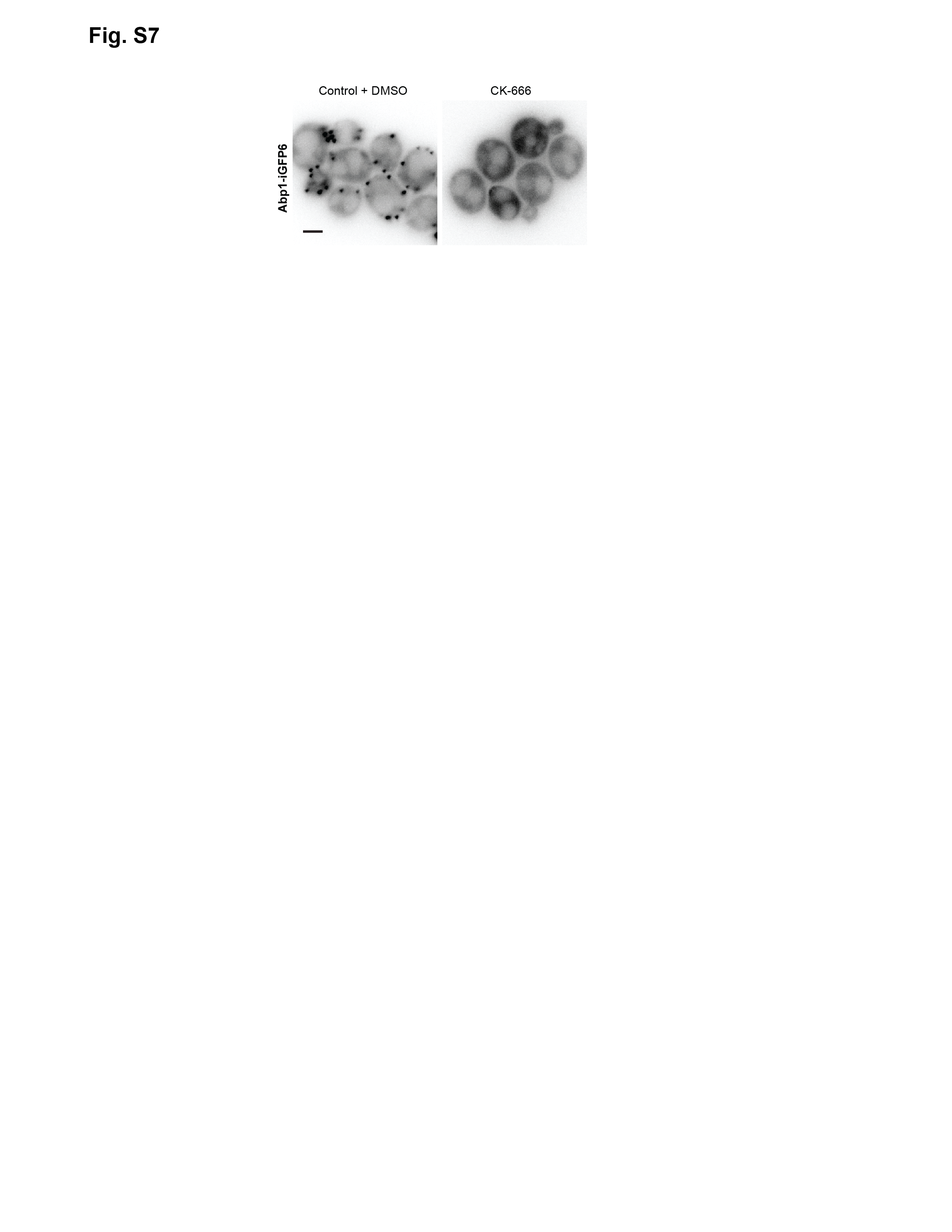
**

**Figure S7: Inhibition of Arp2/3 complex activity by CK-666 abolishes the formation of endocytic patches**. **A.** Widefield fluorescence micrographs of a budding yeast strain expressing Abp1-iGFPx6. Cells treated with DMSO showed typical patch actin dynamics in contrast to the complete loss of Abp1-GFP puncta when cells are treated with CK-666. Brightness and contrast were adjusted individually for each image to optimize visualization of endocytic actin patches. Scale bar: 2 μm

**
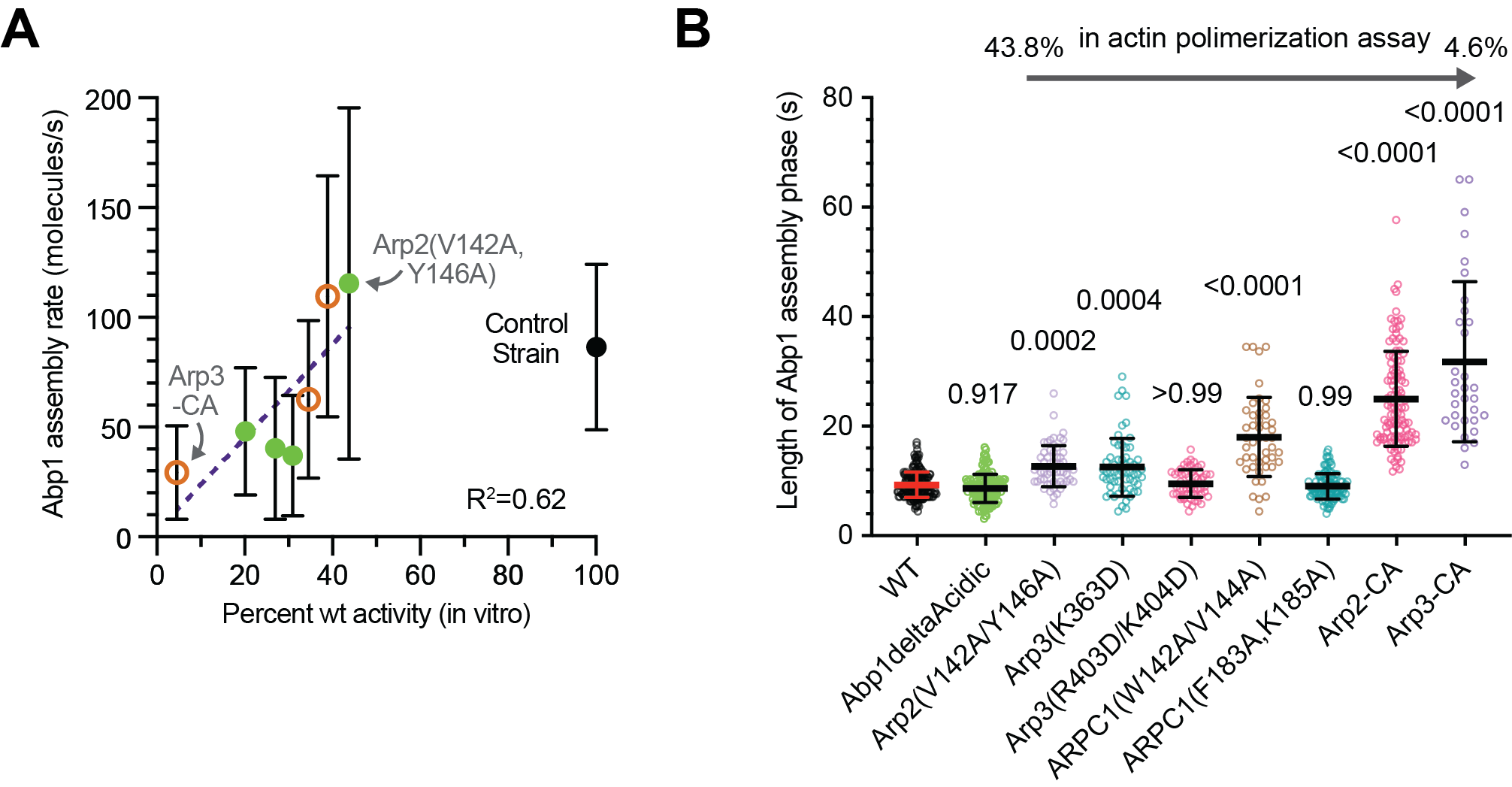
**

**Figure S8: Correlation between in vitro activity of CA-binding site mutants and dynamics of Abp1 at endocytic sites. A.** Plot of the Abp1-TagRFP-T assembly rate versus percent wild-type activity in the pyrene actin polymerization assay using 0.75 µM GST- Las17_281-633_. Arp3 CA binding site mutations are shown as orange circles and Arp2/ARPC1 site mutations are shown as filled green circles. Data were fit to a linear regression with the control strain data point excluded. **B.** Plot of the length of the Abp1-TagRFP-T assembly phase for control and mutant strains. Mutants are listed in order of their activity in the pyrene actin polymerization assay. Statistical significance was measured with an ordinary one-way ANOVA with p values for comparison to the control strain calculated with a Dunnett’s test.

**
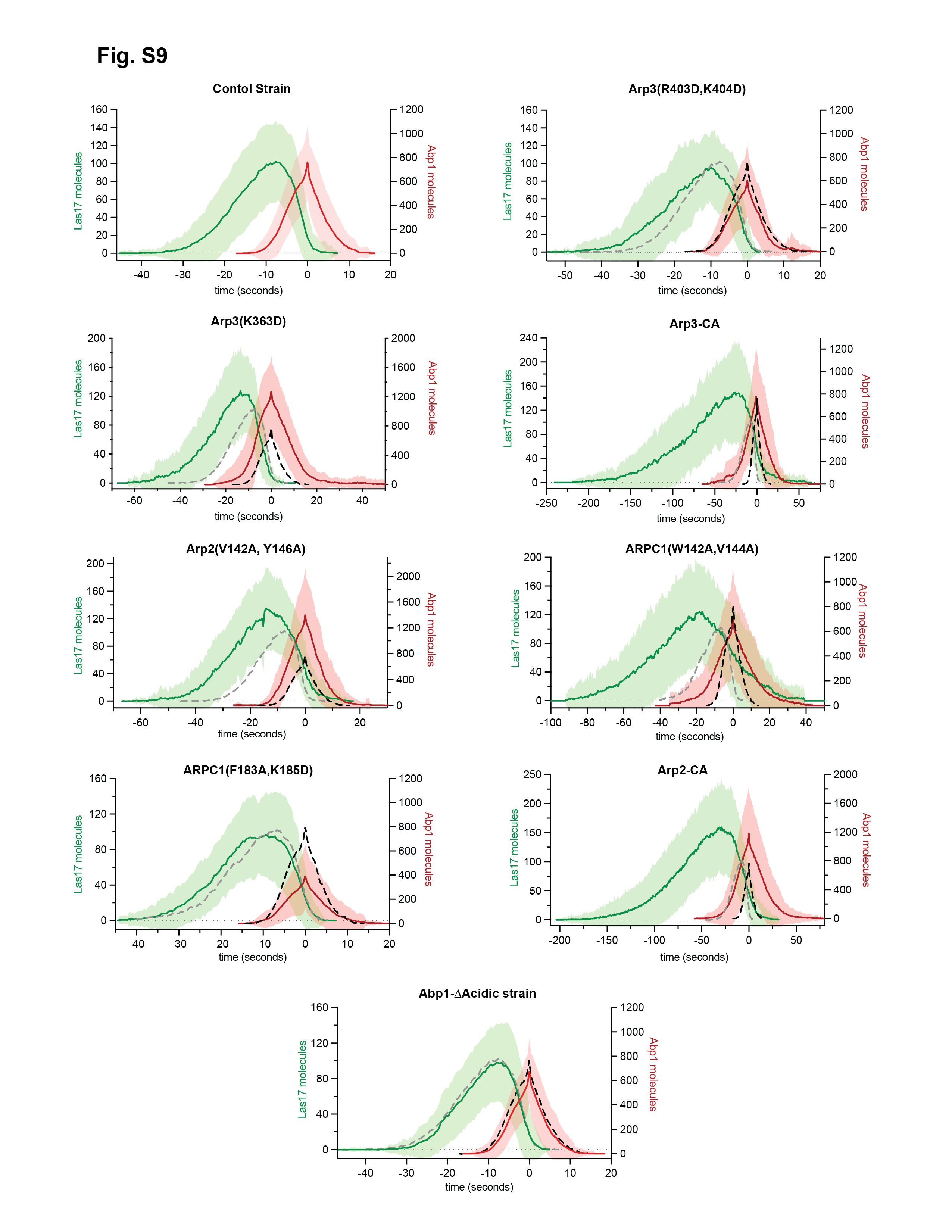
**

**Figure S9: Dynamics of Las17 and Abp1 at endocytic sites.** Plots show the average number of molecules of mNG-Las17 or Abp1-TagRFP-T at endocytic sites as a function of time. Paired fluorescence trajectories were aligned (t=0) to the maximum Abp1-TagRFP-T signal to calculate average. The intensity values were converted to numbers of molecules assuming that the maximum signal for each channel corresponds to 800 and 102 molecules of Abp1 and Las17, respectively (1). Shaded region indicates the standard deviation (n=42-195 individual trajectories). The intensity time courses for Abp1-TagRFP-T and mNG-Las17 in the wild type strain are shown as dashed lines in plots for the mutant strains.


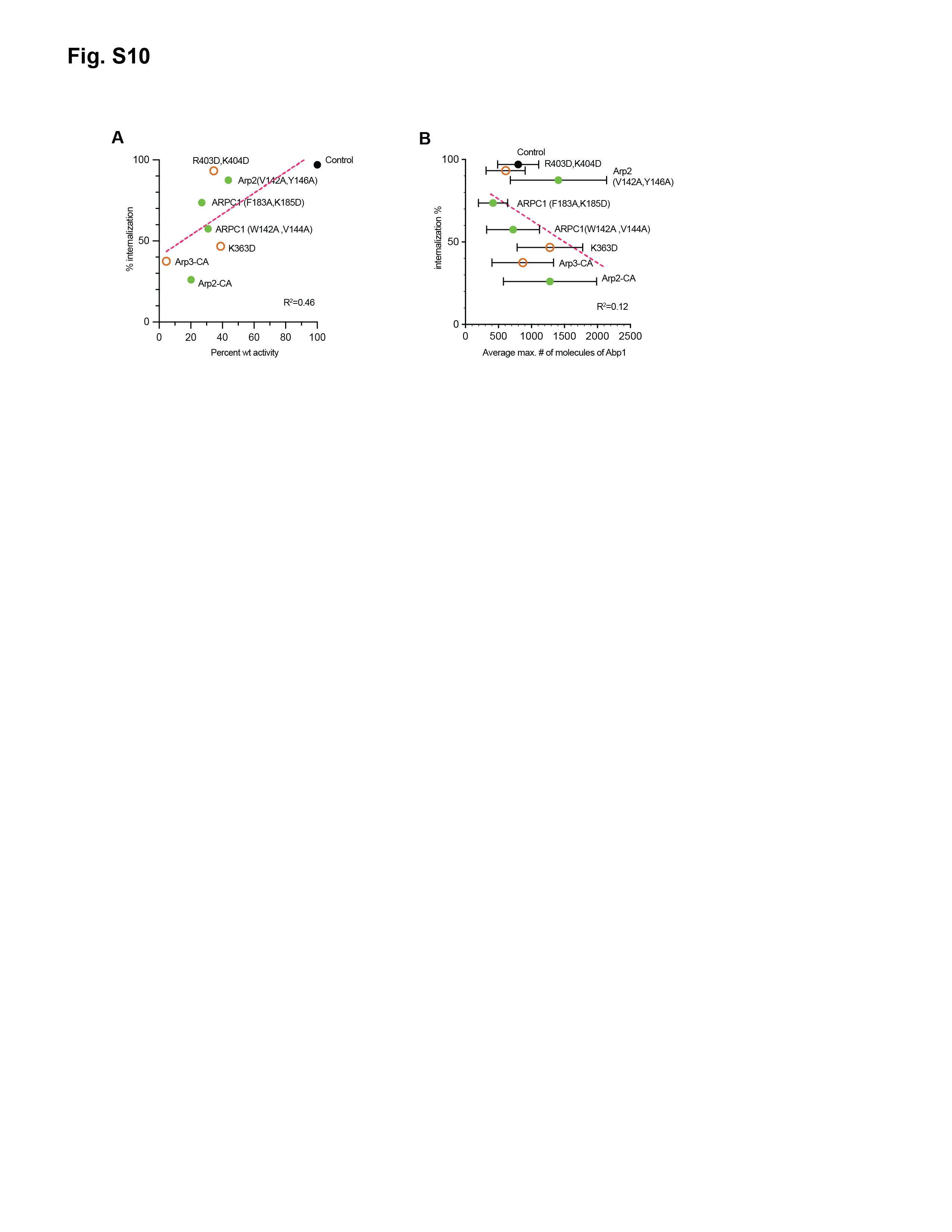


**Figure S10: Assessment of correlation of in vitro Arp2/3 complex activity or the number of Abp1 molecules recruited to endocytic sites with endocytic internalization efficiency. A.** Plot of percent internalization versus percent wild type Arp2/3 complex activity in the pyrene actin polymerization assay with 0.75 µM GST- Las17_281-633_ in control and mutant strains. Arp3 CA binding site mutations are shown as orange circles and Arp2/ARPC1 site mutations are shown as filled green circles. **B.** Plot of percent internalization versus the average maximum number of Abp1-TagRFP-T molecules at endocytic sites in control and mutant strains. For figures A and B, data were fit to a linear regression.

**
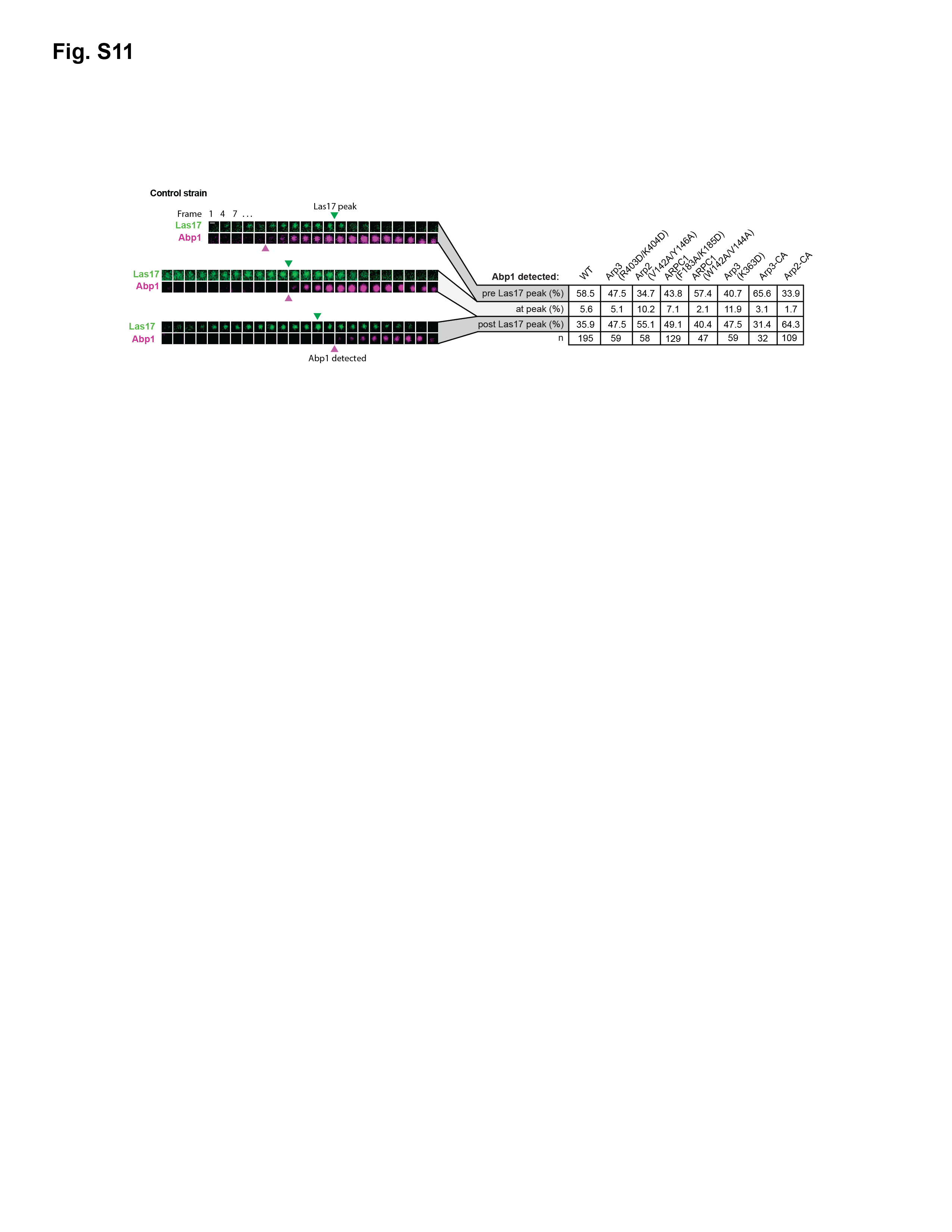
**

**Figure S11: Las17 begins deaccumulation before Abp1 is detectable in some endocytic events.**

Table showing the percentage of endocytic events in which Abp1-TagRFP-T began to accumulate before, after, or at the same time as the peak number of molecules of mNG-Las17 was reached in the mutant and control strains. Montages on the left show one example of each category of event in the control strain. Note that only one of every three frames is shown. Scale bar: 0.3 µm.

**
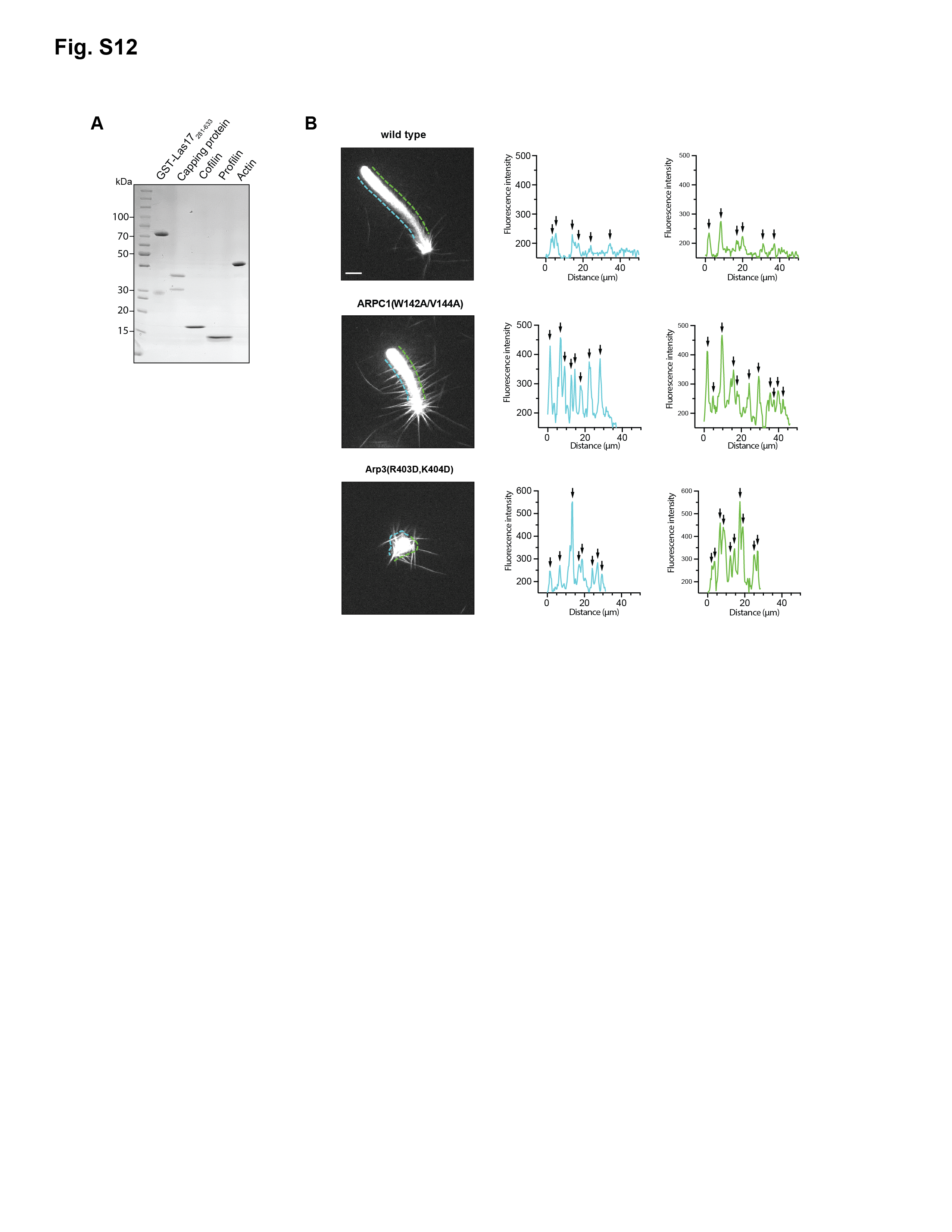
**

**Figure S12: Point mutations at CA binding sites increase actin filament bundling. A.** SDS-PAGE gel of purified proteins used in bead motility assay. **B.** (left) Representative widefield fluorescence microscopy images with manually drawn lines used to measure actin filament bundle intensity. Scale bar: 10 µm. Fluorescence values along the lines were plotted to identify peaks representing each bundle (right, see methods for peak selection procedure). The maximum fluorescence intensity from each peak (black arrows) was used to generate Fig. 7B.

**Table S1:** *S. cerevisiae* strains used in this study

| Table S1: *S. cerevisiae* strains used in this study | | | |
| --- | --- | --- | --- |
| Strain | Protein | Genotype | Reference |
| ScBN114 | wildtype Arp2/3 complex | MATα, ARP3::URA3, his3, leu2, LYS2, ∆arp3::HIS3 | (2) |
| ScBN120 | Arp3 K363D | MATa, ARP3(K363D)::URA3, his3, leu2, ∆arp3::HIS3 | (2) |
| ScBN128 | Arp3 R371D | MATa, ARP3(R371D)::URA3, his3, leu2, ∆arp3::HIS3 | (2) |
| ScBN147 | Arp3 R403D/K404D | MATα, ARP3(R403D, K404D)::URA3, his3, leu2, LYS2, ∆arp3::HIS3 | (2) |
| ScBN150 | Arp3 W191A/T192A | MATα, ARP3(W191A, T192A)::URA3, his3, leu2, lys2, ∆arp3::HIS3 | (2) |
| ScBN151 | ARPC1 K146D/H147A | MATa, ∆arc40::TRP1, ARC40(K146D, H147A)::URA3, his3, trp1, lys2 | (2) |
| ScBN153 | ARPC1 F183A/K185D | MATα, ∆arc40::TRP1, ARC40(F183A, K185D)::URA3, his3, trp1, lys2 | (2) |
| ScBN157 | ARPC1 W142A/V144A | MATα, ∆arc40::TRP1, ARC40(W142A, V144A)::URA3, his3, trp1, lys2 | (2) |
| ScBN200 | Arp2 V142A/Y146A | MATa, ARP2(V142A, Y146A)::URA, his3∆200, leu2-3,112, ∆arp2::HIS3 | (2) |
| ScBN203 | Arp2 K298D | MATα, ARP2(K298D)::URA, his3∆200, leu2-3,112, lys2-801(oc), ∆arp2::HIS3 | (2) |
| ScBN315 | Arp2-CA | MATa, Arp2-CA::URA3, his3∆200, leu2-3,112, ∆arp2::HIS3, ABP1::EGFP_NAT | (2) |
| ScBN323 | Arp3-CA | MATa, Arp3-CA::URA3, his3-∆200, leu2-3, ∆arp3::HIS3, ABP1::EGFP_NAT | (2) |
| ScBN399 | wild type Arp2/3 complex + ARPC2 His-TS tag | MATα, ARP3::URA3, his3, leu2, ∆arp3::HIS3, ARPC2-TEV-HisTag-TwinStrepTag:HygMX6 | this study |
| ScBN402 | Arp3 K363D + ARPC2 His-TS tag | MATa, ARP3(K363D)::URA3, his3, leu2, ∆arp3::HIS3, ARPC2-TEV-His12-TwinStrep:HygMX6 | this study |
| ScBN403 | Arp3 R371D + ARPC2 His-TS tag | MATa, ARP3(R371D)::URA3, his3, leu2, ∆arp3::HIS3,ARPC2-TEV-His12-TwinStrep:HygMX6 | this study |
| ScBN404 | Arp3 R403D/K404D + ARPC2 His-TS tag | MATα, ARP3(R403D, K404D)::URA3, his3, leu2, ∆arp3::HIS3, ARPC2-TEV-His12-TwinStrep:HygMX6 | this study |
| ScBN405 | ARPC1 F183A/K185A + ARPC2 His-TS tag | MATα, ∆arc40::TRP1, ARC40(F183A, K185D)::URA3, his3, trp1, lys2, ARPC2-TEV-His12-TwinStrep:HygMX6 | this study |
| ScBN406 | Arp2 V142A/Y146A + ARPC2 His-TS tag | MATa, ARP2(V142A, Y146A)::URA, his3∆200, leu2-3,112, ∆arp2::HIS3, ARPC2-TEV-His12-TwinStrep:HygMX6 | this study |
| ScBN407 | Arp2 K298D + ARPC2 His-TS tag | MATα, ARP2(K298D)::URA, his3∆200, leu2-3,112, lys2-801(oc), ∆arp2::HIS3, ARPC2-TEV-His12-TwinStrep:HygMX6 | this study |
| ScBN408 | Arp3-CA + ARPC2 His-TS tag | MATa, Arp3-CA::URA3, ARPC2-TEV-His-TwinStrep::hphMX6, ∆arp3::HIS3, ABP1::EGFP_NAT | this study |
| ScBN411 | Arp3 W191A/T192A + ARPC2 His-TS tag | MATα, ARP3(W191A, T192A)::URA3, his3, leu2, lys2, ∆arp3::HIS3. ARPC2-TEV-His12-TwinStrep:HygMX6 | this study |
| ScBN413 | ARPC1 K146D/H147A + ARPC2 His-TS tag | MATa, ∆arc40::TRP1, ARC40(K146D, H147A)::URA3, his3, trp1, lys2, ARPC2-TEV-His12-TwinStrep:HygMX6 | this study |
| ScBN414 | ARPC1 W142A/V144A + ARPC2 His-TS tag | MATα, Darc40::TRP1, ARC40(W142A, V144A)::URA3, his3, trp1, lys2, ARPC2-TEV-His12-TwinStrep:HygMX6 | this study |
| ScBN425 | Arp2-CA + ARPC2 His-TS tag | MATa, Arp2-CA::URA3, his3∆200, leu2-3,112, ∆arp2::HIS3, ABP1::EGFP_NAT, ARPC2-TEV-His-TwinStrep::hphMX6 | this study |
| ScBN369 | wild type complex, mNG-Las17, ABP1-TagRFP-T | MATa, ARP3::URA3, his3, leu2, ∆arp3::HIS3, natMX6::mNeonGreen-Las17, ABP1-TagRFP-T::kanMX6 | this study |
| ScBN426 | Arp3 R403D/K404D, mNG-Las17, ABP1-TagRFP-T | MATα, ARP3(R403D, K404D)::URA3, his3, leu2, ∆arp3::HIS, ABP1-TagRFP-T::kanMX6, natMX6::mNeonGreen-Las17 | this study |
| ScBN362 | Arp3-CA, mNG-Las17, ABP1- TagRFP-T | MATa, Arp3-CA::URA3, his3, ∆arp3::HIS3, ABP1-TagRFP-T::kanMX6, natMX6::mNeonGreen-Las17 | this study |
| ScBN356 | Arp3 K363D, mNG-Las17, ABP1- TagRFP-T | MATa, ARP3(K363D)::URA3, his3, leu2, ∆arp3::HIS3, natMX6::mNeonGreen-Las17, ABP1-TagRFP-T::kanMX6 | this study |
| ScBN363 | Arp2-CA, mNG-Las17, ABP1- TagRFP-T | MATa, Arp2-CA::URA3, his3∆200, leu2-3,112, ∆arp2::HIS3, natMX6::mNeonGreen-Las17, ABP1-TagRFP-T::kanMX6 | this study |
| ScBN354 | Arp2 V142A/Y146A, mNG-Las17, ABP1- TagRFP-T | MATa, ARP2(V142A, Y146A)::URA, his3∆200, leu2-3,112, ∆arp2::HIS3, natMX6::mNeonGreen-Las17, ABP1-TagRFP-T::kanMX6 | this study |
| ScBN357 | ARPC1 W142A/V144A, mNG-Las17, ABP1- TagRFP-T | MATα, ARC40::TRP1, ARC40(W142A, V144A)::URA3, his3, trp1, lys2, natMX6::mNeonGreen-Las17, ABP1-TagRFP-T::kanMX6 | this study |
| ScBN429 | ARPC1 F183A/K185D, mNG-Las17, ABP1- TagRFP-T | MATα, ∆arc40::TRP1, ARC40(F183A, K185D)::URA3, his3, trp1, lys2, ABP1-TagRFP-T::kanMX6, natMX6::mNeonGreen-Las17 | this study |
| ScBN462 | wild type complex, mNG-Las17, ABP1-∆Acidic- TagRFP-T | MATa, ARP3::URA3, his3, leu2, ∆arp3::HIS3, natMX6::mNeonGreen-Las17, ABP1-∆Acidic-TagRFP-T::kanMX6 | this study |

**Video S1.**  Widefield fluorescence microscopy videos showing control or Arp3 binding site mutants *S. cerevisiae* cells expressing mNG-Las17 (green) and Abp1-Tag-RFP-T (magenta). Scale bar: 2 µm.

**Video S2.** Widefield fluorescence microscopy videos showing control or Arp2/ARPC1 binding site mutants *S. cerevisiae* cells expressing mNG-Las17 (green) and Abp1-Tag-RFP-T (magenta). Scale bar: 2 µm.

**Video S3.** Representative fluorescence microscopy videos of reconstituted actin assembly assays containing WT Arp2/3 complex or Arp3 binding site mutants. Scale bar: 20 µm.

**Video S4.** Representative fluorescence microscopy videos of reconstituted actin assembly assays containing WT Arp2/3 complex or Arp2/ARPC1 binding site mutants. Scale bar: 20 µm.

**Video S5.** Representative fluorescence microscopy videos of reconstituted actin assembly assays with the Arp-CA fusions or in absence of Arp2/3 complex. Scale bar: 20 µm.

**References**

1. Sun, Y., Schöneberg, J., Chen, X., Jiang, T., Kaplan, C., Xu, K., Pollard, T. D., and Drubin, D. G. (2019) Direct comparison of clathrin-mediated endocytosis in budding and fission yeast reveals conserved and evolvable features. *Elife*. **8**, e50749

2. Luan, Q., Zelter, A., MacCoss, M. J., Davis, T. N., and Nolen, B. J. (2018) Identification of Wiskott-Aldrich syndrome protein (WASP) binding sites on the branched actin filament nucleator Arp2/3 complex. *Proc. Natl. Acad. Sci. U.S.A.* **115**, E1409–E1418
